# Determining Critical Topics for Undergraduate Surgical Education in Rwanda: Results of a Modified Delphi Process and a Consensus Conference

**DOI:** 10.7759/cureus.43625

**Published:** 2023-08-17

**Authors:** Barnabas T Alayande, Callum W Forbes, Jules Iradakunda, Jean Paul Majyambere, Matthew T Hey, Brittany L Powell, Juliana Perl, Natalie McCall, Tomlin Paul, JC Allen Ingabire, Natnael Shimelash, Emmanuel Mutabazi, Emmanuel O Kimto, Gambo Musa Danladi, Ronald Tubasiime, Jennifer Rickard, Claire Karekezi, Gabriel Makiriro, Simon Pierre Bigirimana, James G Harelimana, Ahmed ElSayed, Alain Jules Ndibanje, Christophe Mpirimbanyi, Ornella Masimbi, Mick Ndayishimiye, Frederick Ntabana, Billy Thomson Haonga, Geoffrey A Anderson, Jean Claude Byringyiro, Faustin Ntirenganya, Robert R Riviello, Abebe Bekele

**Affiliations:** 1 General Surgery, Center for Equity in Global Surgery, University of Global Health Equity, Kigali, RWA; 2 Global Health and Social Medicine, Program in Global Surgery and Social Change, Harvard Medical School, Boston, USA; 3 Global Health and Population, Harvard School of Public Health, Boston, USA; 4 Anesthesiology, Center for Equity in Global Surgery, University of Global Health Equity, Kigali, RWA; 5 School of Medicine, Center for Equity in Global Surgery, University of Global Health Equity, Kigali, RWA; 6 Surgery, Butaro District Hospital, Kigali, RWA; 7 Surgery, Center for Equity in Global Surgery, University of Global Health Equity, Kigali, RWA; 8 Surgery, Center for Surgery and Public Health, Brigham and Women’s Hospital, Boston, USA; 9 Biodesign, Center for Equity in Global Surgery, University of Global Health Equity, Kigali, RWA; 10 Division of Clinical Medicine, University of Global Health Equity, Kigali, RWA; 11 Educational Development and Quality Center, University of Global Health Equity, Kigali, RWA; 12 Surgery, School of Medicine and Pharmacy, College of Medicine and Health Sciences, University of Rwanda, Kigali, RWA; 13 Research Unit, Surgical Equity and Research Centre, Jos, NGA; 14 Surgery, Kibogora Hospital, Kibogora, RWA; 15 Surgery, University of Minnesota, Minneapolis, USA; 16 Surgery, Neurosurgery Unit, Rwanda Military Hospital, Kigali, RWA; 17 Surgery, Alzaiem Alazhari University, Khartoum, SDN; 18 Surgery, University Teaching Hospital of Kigali, Kigali, RWA; 19 Surgery, Kibagabaga Level II Teaching Hospital, Kigali, RWA; 20 Simulation, Center for Equity in Global Surgery, University of Global Health Equity, Kigali, RWA; 21 Orthopaedic Surgery, Muhimbili University of Health and Allied Sciences, Dar es Salaam, TZA; 22 Trauma, Burns, and Critical Care, Center for Equity in Global Surgery, University of Global Health Equity, Kigali, RWA; 23 Orthopedics, University Teaching Hospital of Kigali, Kigali, RWA; 24 NIHR Research Hub on Global Surgery, University of Rwanda, Kigali, RWA; 25 Surgery, Center for Surgery and Public Health, Brigham and Women’s Hospital, Kigali, RWA; 26 Cardiothoracic Surgery, Center for Equity in Global Surgery, University of Global Health Equity, Kigali, RWA

**Keywords:** medical school, rwanda, surgery curriculum, consensus, delphi

## Abstract

Background

Developing a contextually appropriate curriculum is critical to train physicians who can address surgical challenges in sub-Saharan Africa. An innovative modified Delphi process was used to identify contextually optimized curricular content to meet sub-Saharan Africa and Rwanda’s surgical needs.

Methods

Participants were surgeons from East, Central, Southern, and West Africa and general practitioners with surgical experience. Delphi participants excluded or prioritized surgical topic areas generated from extensive grey and formal literature review. Surgical educators first screened and condensed identified topics. Round 1 screened and prioritized identified topics, with a 75% consensus cut-off based on the content validity index and a prioritization score. Topics that reached consensus were screened again in round 2 and re-prioritized, following controlled feedback. Frequencies for aggregate prioritization scores, experts in agreement, item-level content validity index, universal agreement and scale-level content validity index based on the average method (S-CVI/Ave) using proportion relevance, and intra-class correlation (ICC) (based on a mean-rating, consistency, two-way mixed-effects model) were performed. We also used arithmetic mean values and modal frequency. Cronbach's Alpha was also calculated to ascertain reliability. Results were validated through a multi-institution consensus conference attended by Rwanda-based surgical specialists, general practitioners, medical students, surgical educators, and surgical association representatives using an inclusive, participatory, collaborative, agreement-seeking, and cooperative, *a priori* consensus decision-making model.

Results

Two-hundred and sixty-seven broad surgical content areas were identified through the initial round and presented to experts. In round 2, a total of 247 (92%) content areas reached 75% consensus among 31 experts. Topics that did not achieve consensus consisted broadly of small intestinal malignancies, rare hepatobiliary pathologies, and transplantation.

In the final round, 99.6% of content areas reached 75% consensus among 31 experts. The highest prioritization was on wound healing, fluid and electrolyte management, and appendicitis, followed by metabolic response, infection, preoperative preparation, antibiotics, small bowel obstruction and perforation, breast infection, acute urinary retention, testicular torsion, hemorrhoids, and surgical ethics. Overall, the consistency and average agreement between panel experts was strong. ICC was 0.856 (95% CI: 0.83-0.87). Cronbach's Alpha for round 2 was very strong (0.985, 95% CI: 0.976-0.991) and higher than round 1, demonstrating strong reliability. All 246 topics from round 4 were verbally accepted by 40 participants in open forum discussions during the consensus conference.

Conclusions

A modified Delphi process and consensus were able to identify essential topics to be included within a highly contextualized, locally driven surgical clerkship curriculum delivered in rural Rwanda. Other contexts can use similar processes to develop relevant curricula.

## Introduction

Low- and middle-income countries (LMICs) experience a deficit of approximately 143 million surgical procedures each year, and surgical provider shortfalls contribute significantly to this huge gap in access to care [1]. These deficits are global but are more pronounced in sub-Saharan Africa (SSA) [2,3]. While the recommended surgeon, anesthetist, and obstetrician (SAO) density is 20 per 100,000 population, SSA has less than two surgical specialists per 100,000. The challenge is even more pronounced in Eastern, Central, and Southern Africa which has only 0.53 surgeons per 100,000 population [2,3]. Training of surgical providers needs to be prioritized in attempting to meet such SAO provider density targets.

Between 2016 and 2019, 24 Rwandan surgeons were certified by the College of Surgeons of Eastern, Central, and Southern Africa (COSECSA) [4]. In addition, several specialist trainees were certified through the University of Rwanda [5] and not-for-profit training programs like the Pan African Association of Christian Surgeons [6]. However, current surgical specialist volumes in Rwanda are still insufficient to handle the local surgical burden of disease of about 12,000 surgical conditions per 100,000 people, which is about 27,160 surgeries required per qualified surgeon in the country [7,8]. The role of non-specialist physicians in providing basic, life-saving surgical care in SSA is already established [9-11]. In Rwanda, general practitioners (in this context, medical doctors with no specialist residency training) in rural district hospitals perform most of the basic general surgery procedures, cesarean sections, and closed fracture manipulation [12]. As such, expectations and training needs for non-specialist doctors in LMICs differ from those in high-income countries (HICs) as there is a need to prepare medical graduates entering into general practice in resource-constrained locations for essential surgical procedures [9,10,13]. However, it has been observed that LMIC surgical training curricula are often simply adopted from HIC institutions with little alteration [14].

The University of Global Health Equity (UGHE) is a new health sciences university based in rural Rwanda [15]. The overall vision for surgical training at UGHE is to equip medical students with the necessary skills to carry out Bellwether surgical procedures (cesarean section, laparotomy, and management of open fractures) within the scope of their practice [16], manage surgical emergencies, and appropriately refer surgical patients to higher levels of care when required. The University of Rwanda is the key player in the training of medical practitioners and specialists required to meet the country’s surgical needs. It is the largest public training and research institution in Rwanda [17]. Both institutions collaborated to define and prioritize topics for undergraduate surgical training for their context. We describe a modified Delphi consensus process used to derive topical core surgical content areas to be used in curriculum development for undergraduate medical students in Rwanda.

The abstract of preliminary results of this work was submitted to the American College of Surgeons Scientific Conference on October 2022, presented at the 2022 Rwanda Surgical Society Annual Conference on November 19, 2022, and presented at the 2022 College of Surgeons of East, Central, and Southern Africa Scientific Conference on December 8, 2022.

Aims of the study were to derive topical core curriculum content areas to be used in curriculum development for undergraduate surgery trainees in SSA and Rwanda specifically, to identify priorities that will be relevant to local surgical practice and for preparing trainees for relevant rural surgical practice, and to generate consensus on undergraduate surgery core curriculum content for Rwanda.

## Materials and methods

With limited precedent regarding the development and delivery of contextualized undergraduate surgical curricula across SSA, there was a need to generate expert consensus on the topic. The Delphi technique is a well-established approach with three key characteristics that can be used to answer a research question, based on the consensus of subject matter experts [18]. Firstly, it is based on a series of rounds. Questions in each subsequent round are based on findings from the previous round, and the study evolves in response to earlier findings. Next, respondents can see the results of the previous rounds in order to permit them to reflect on others’ views and possibly reconsider their own. Finally, results of each round are shared anonymously to avoid bias [18]. A modified Delphi process was used to develop expert consensus as there is consistent evidence to support the superiority of group decision-making over individual opinion when seeking expert judgment [19]. Of methods utilized to identify consensus and solicit group opinion, we selected a modified Delphi technique over nominal techniques of a simple survey or a strict consensus conference [20]. This technique permits the views of experts to be sought and combined without them necessarily having to meet; this was preferable considering the constraints of distance, time, and COVID restrictions.

We modified the Delphi technique in two ways: by developing a questionnaire for circulation to panelists based on a list obtained through a rigorous review of the literature (confirmatory approach), as opposed to the generation of the questionnaire by an expert Delphi panel, and by permitting new Rwanda-based panelists to join in the second round. We also combined inclusion/exclusion and prioritization (traditionally separate rounds) in each consolidated round. This confirmatory approach to the first round has been utilized in several studies through which content for undergraduate curricula in other areas has been obtained [21-23]. This study was guided by the Guidance on Conducting and REporting DElphi Studies (CREDES) recommendations [24].

**Figure 1:**
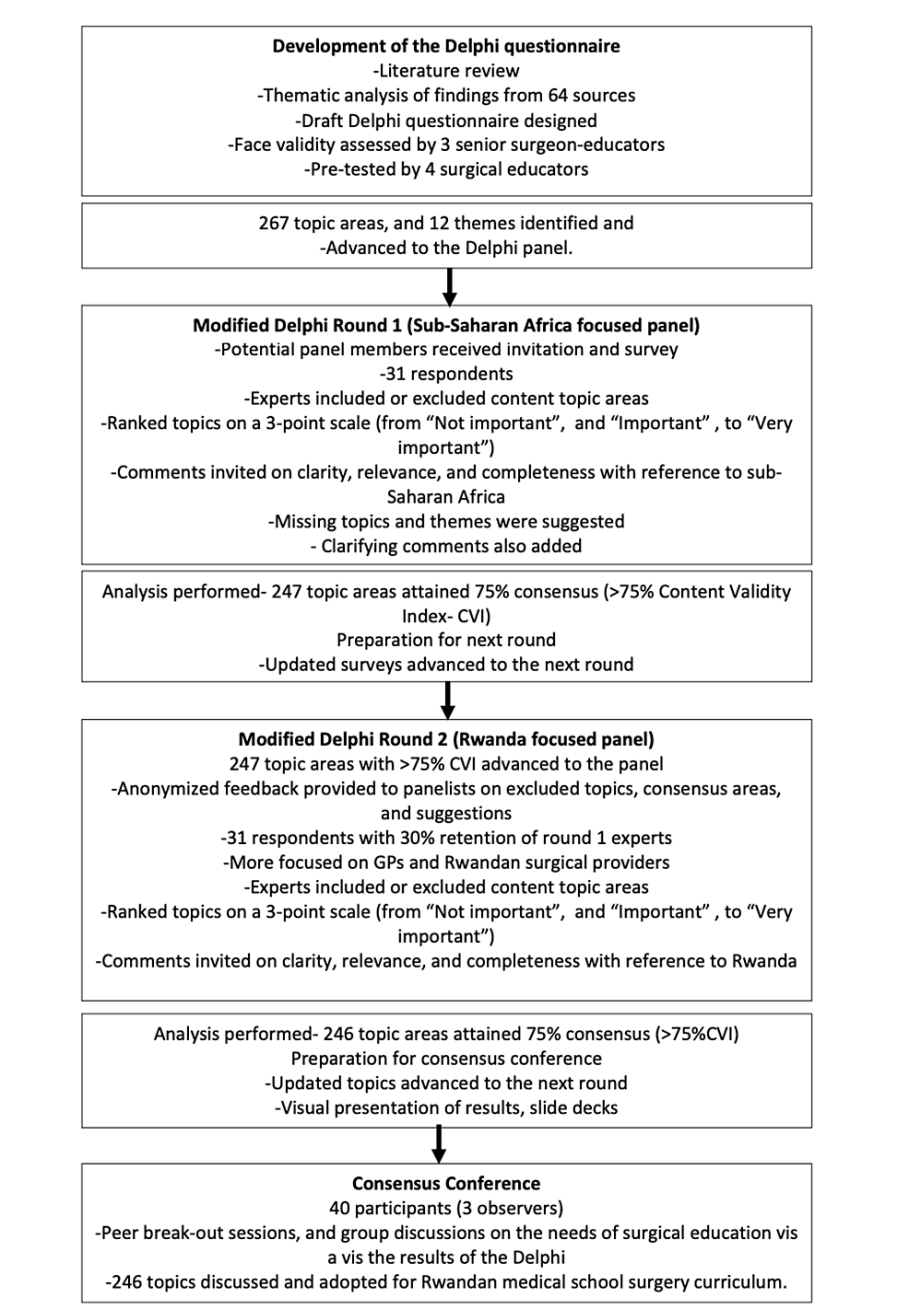
Course of the Consensus Study

Following the generation of the topic list through a literature review, two consolidated rounds of consensus surveying were undertaken followed by an in-person consensus conference. The course of this multi-step consensus study is demonstrated in Figure 1.

Literature review and thematic analysis/topic selection

**Figure 2:**
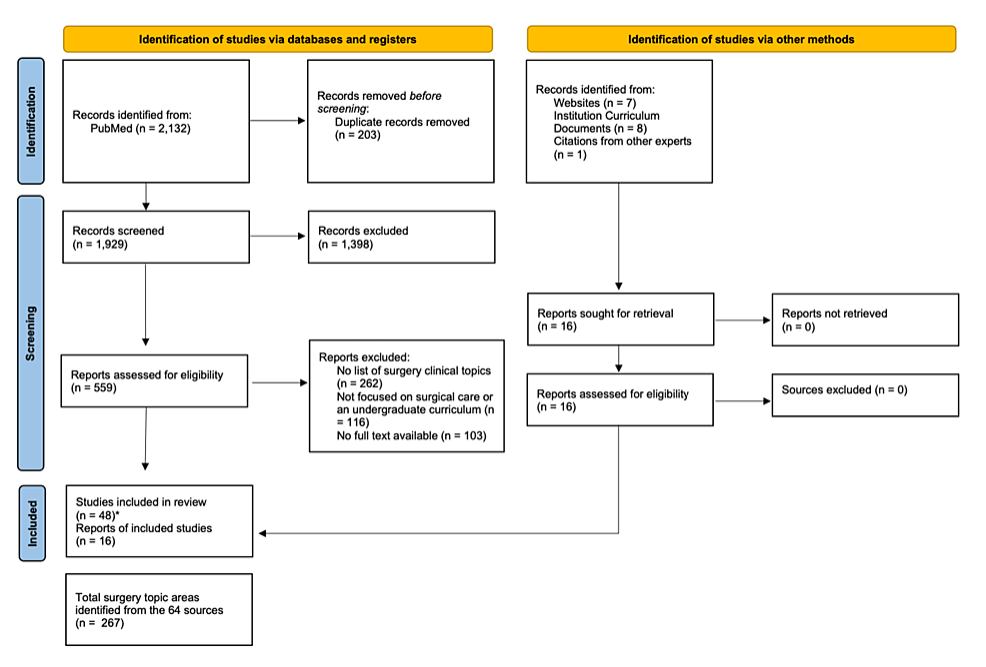
PRISMA Flow Diagram PRISMA: Preferred Reporting Items for Systematic Reviews and Meta-Analyses

Development of the Delphi questionnaires followed the process of literature review, thematic/topical analysis, and expert validation. Investigators (BA, EOK, GMD) conducted a wide scoping review of existing grey literature including available existing curricula and specific surgical training websites (Appendix 1). Curricula from SSA, North America, Asia, and Europe were consulted in this topic search. Potentially relevant surgical topics were also collected using a PubMed search for surgical curriculum-related papers for medical students. Key search terms used were “Surgery AND (Education, Medical, Undergraduate OR Clinical Clerkship)) AND Students, Medical AND Curriculum NOT (Obstetrics OR Gynecology OR Anatomy).” The database search was limited to 20 years (Appendix 1). A summary of the search is shown in Figure 2. Results of the database search are found in Appendix 2.

Identifying, screening, and consolidating surgical topics

Topic areas derived from the literature search were reviewed by a study committee of five clinical surgery experts and surgical educators (AB, RR, GA, BA, and LI). Several topics were combined, removed, and/or rearranged based on considerations of applicability to African clinical general practice and feasibility with an aim to present as many topic areas as possible to the expert panel. The surgical knowledge topic areas were placed into 12 broad domains (Appendix 3). The Delphi questionnaire was then revised by three senior surgeon-educators to establish both content face validity and clarity. The survey questionnaire was then pretested for field clarity, time for completion, and general usability by four separate surgical educators following which the committee further revised and refined the electronic survey instrument.

Delphi participant selection

Purposive sampling was used to select experts including both surgical specialists and non-specialist surgical care providers familiar with the Rwandan, East Africa, and SSA surgical contexts. Delphi participants were identified through the Rwandan Surgical Society and the COSECSA. We aimed to purposively include a minimum of 23 experts, targeting surgical providers including African specialist surgeons and non-specialist surgical providers [25].

Our definition of expert providers, by which we invited panelists, included surgeons with more than five years of experience in the Rwandan context, including those working in Rwandan District, provincial, new referral, university, and/or mission hospitals; surgeons practicing in any of the African sub-regions as defined by the African Union [26]; and non-specialist surgical providers (general practitioners) practicing in Rwandan district hospitals, with more than two years of experience.

**Figure 3:**
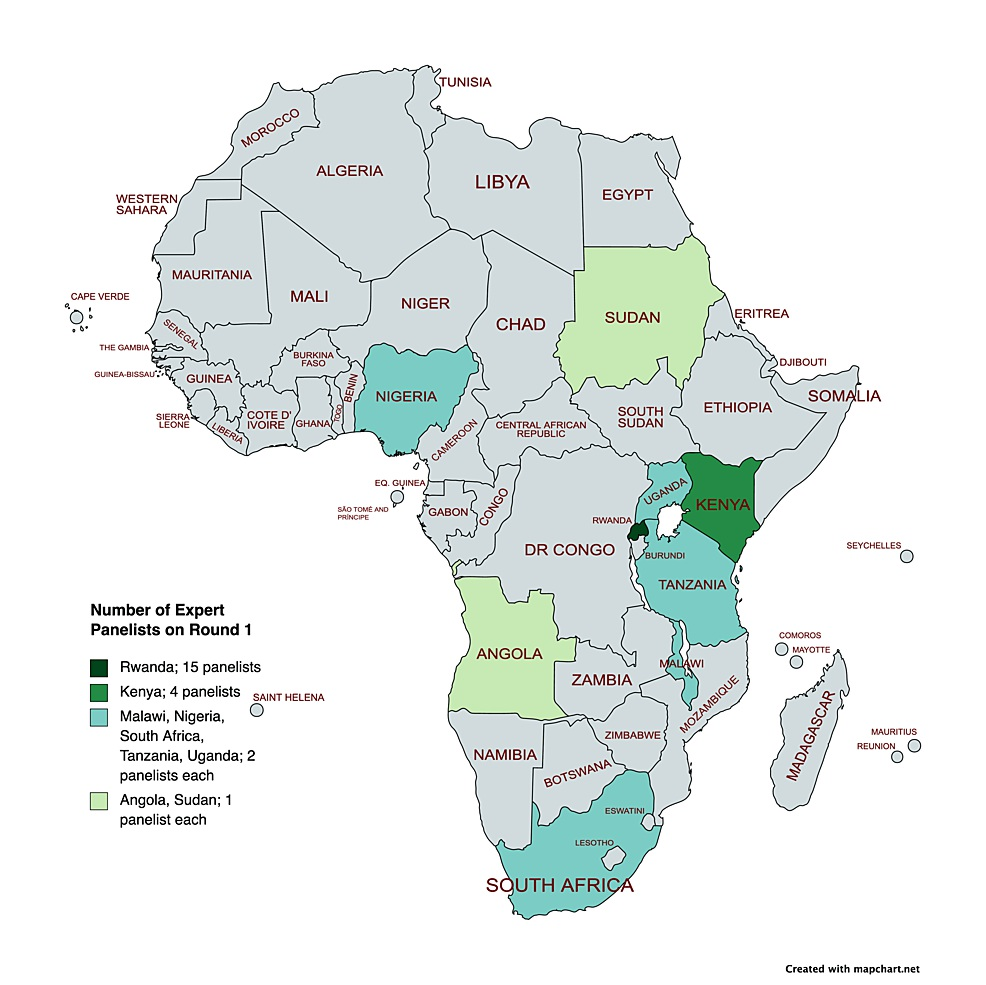
Geographic Distribution of Expert Panelists

Of 320 participant invitations based on the expert inclusion criteria, 31 (10%) surgical providers consented to participate in July 2021. Figure 3 shows the geographic distribution of the expert panel for the first round. Half of them (15) were working in the Rwandan context. The experts included 12 general surgeons (39%), five orthopedic surgeons (16%), four general practitioners (13%), three neurosurgeons (10%), two pediatric surgeons (6%), two plastic surgeons (6%), two cardiothoracic surgeons (6%), one maxillofacial and oral surgeon (3%), and one trauma and acute care surgeon (3%). The male-to-female ratio was approximately 3:1.

For the second round, 87% (27) of the respondents worked in Rwanda. Eleven (35%) respondents were retained from the first round, and the male-to-female ratio was 4:1. Representation of expert general practitioners increased from 13% to 35%.

Modified Delphi round 1

Round 1 was undertaken with the aim of inclusion, exclusion, and prioritization of curriculum topic areas based on applicability to SSA general practice, unique purpose of the training institution, and feasibility in a low-income setting. The content validity index (CVI) was calculated to establish Delphi consensus from the ratings of panel members [27,28]. The CVI describes the percentage of respondents who rated the thematic areas as important or somewhat important. For topics that were included by a respondent, priority ranking (based on whether the topics were not important, somewhat important, or important) was carried out using a calculated aggregate priority score. Prioritization was performed by calculating an average score (in percentage) out of a total of 4 points (exclude = 1, not important = 2, somewhat important = 3, important = 4). Aggregate group prioritization to the level of “somewhat important” was set at a score of 3 out of 4 (75%). A prioritization ranking of 75% was set as the predefined consensus level and was used to determine which topics advanced through to the next round [29]. If a CVI >75% or aggregate prioritization score of >75% was attained, consensus was assumed. Finally, respondents were given the opportunity to suggest additional subject areas, not already included in the survey, that they felt should be incorporated into an undergraduate curriculum. Prioritized topics were advanced to the next round. Consistency and average agreement between panel experts were calculated. Intra-class correlation (ICC) was calculated using Shrout and Fleiss 1979 Convention 3,k consistency, and a two-way mixed-effects model. Cronbach's Alpha was also calculated to ascertain reliability.

Modified Delphi round 2

Consensus items were advanced to the next modified Delphi round from March to August 2022 to establish consensus between 31 respondents. Participants were given anonymized feedback on the topics that did and did not achieve consensus from the first round and were permitted to comment on these. Inclusion, exclusion, and re-prioritization were repeated for prioritized topics from the first round. A CVI of at least 75% was set as the predefined consensus level and used to determine which topics advanced through to the consensus conference. If this level was attained, consensus for this round was assumed, and further validation was not deemed necessary. Free text responses were encouraged to add unique perspectives to the round. Results from the second round were advanced to the Consensus conference. ICC and Cronbach's Alpha were also calculated.

Consensus conference

The consensus conference was held on 12th August 2022 and incorporated input from a broader range of 40 stakeholders. Those who participated included surgical educators, specifically the Dean, Heads of Surgery Departments, and Module Leaders/Clerkship Directors from each of the medical schools based in Rwanda. Furthermore, representatives of the Rwanda Surgical Society, Rwanda Medical and Dental Council, representatives of District Hospital Leadership, and Rwandan surgeons from rural communities were also invited. To ensure a balanced panel, experienced General Practitioners with greater than two years of experience in providing non-specialist surgical care, interns, and recent medical graduates also contributed to the confirmatory stakeholder conference. The conference was co-chaired by the Heads of Surgery of the University of Rwanda and the University of Global Health Equity.

**Figure 4:**
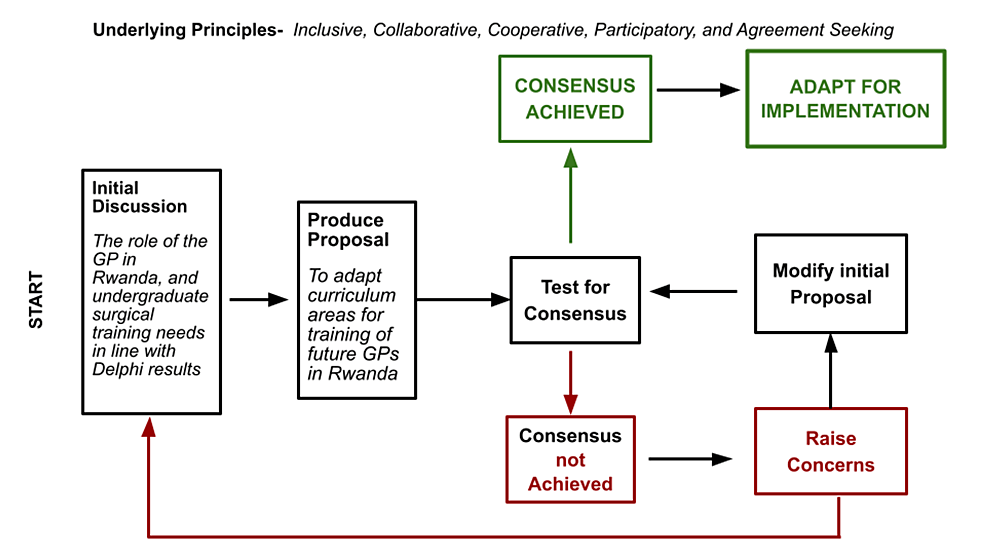
Consensus Decision-Making Model Adopted for the Curriculum Consensus Conference

An inclusive, participatory, collaborative, agreement-seeking, and cooperative, *a priori* consensus decision-making model was adopted based on the process in Figure 4 [30].

The consensus conference entailed open discussion of the Delphi results. Participants were presented with the results from prior rounds of the Delphi process and asked to state their agreement or disagreement with the consensus topics following homogeneous small group discussions (as surgical educators versus General Practitioners, recent graduates from medical school, and medical students) about the state of medical education in Rwanda and the role of the non-medical specialist. A decision rule of unanimous agreement for the inclusion of any topic area was applied. Participants were encouraged to ask clarifying questions, block consensus where necessary, agree with reservations, or to stand aside with reasons. Furthermore, input and refinements were sought regarding the prioritization or ranking of topic areas. Any curriculum area that did not attain unanimous agreement by all conference participants was to be removed.

Survey administration and data handling

Surveys were developed and completed through secure Google Forms (Google LLC, Mountain View, USA) with results automatically inputted into a Google Sheet (Google LLC, Mountain View, USA) with access limited to those within the core research group. The results for each Delphi round were then downloaded as a Microsoft Excel file and analysis was performed using Microsoft Excel version 16.67 (Microsoft Corporation, Washington, USA) on an encrypted, password-protected device.

Institutional review board approval and participant consent

The Modified Delphi consensus process was approved by the UGHE ethical review committee (UGHE-IRB/2021/059). Informed consent was obtained from all participants at every level of the consensus process. Electronic informed consent was obtained during the Delphi rounds, and written informed consent witnessed by the consensus conference secretariat was obtained from participants at the consensus conference. The participants were informed in writing about the rationale, methods, and aim of the Delphi and consensus conference and had the opportunity to ask clarifying questions at the venue.

## Results

Literature review and thematic analysis/topic selection

From a total of 64 independent sources, a list of 267 surgical topics in 12 broad surgical content areas was identified and compiled for inclusion in the first round Delphi survey. The full list of identified topic areas is available in Appendix 3.

Modified Delphi round 1

From the 267 broad surgical content areas, a total of 247 (92%) attained an aggregated prioritization rating of >75% among 31 experts (Appendix 4). ICC was 0.919 (95% CI: 0.907-0.931), using Shrout and Fleiss 1979 Convention 3,k for 247 subjects and 31 judges/measurements, consistency, and a two-way mixed-effects model. Cronbach’s alpha was 0.984 (95% CI: 0.976-0.990) and demonstrated strong reliability. Topics that did not achieve consensus included small intestinal malignancies, rare hepatobiliary pathologies, and organ transplantation among others (Table 1).

**Table 1:** Prioritization Scores from the First Round of the Modified Delphi Showing Borderline and Excluded Topics

Topic Areas System/Organ (Named Topic)	Aggregate Prioritization Score		Disposition
Abdomen - General / Hernia (Management of Abdominal Wall Tumors )		75.78	Borderline, Included
Abdomen - General / Hernia (Other Hernia (Spigelian, Lumbar, etc.))		75.78	
Esophagus (Esophageal Motility Disorders)		75.78	
Thoracic (Mediastinal Tumors)		75.78	
Hepatobiliary (Biliary Duct Tumors)		75.00	
Hepatobiliary (Gallbladder Cancer)		75.00	
Small Intestine (Small Intestinal Lymphoma)		75.00	
Esophagus (Paraesophageal Hernia)		75.00	
Orthopedic Surgery (Scoliosis)		75.00	
Pancreas (Management of Complex Pancreatitis)*		74.22	*Excluded
Small Intestine (Small Intestinal Carcinoid Tumor)*		74.22	
Thoracic (Benign Lung Tumors)*		74.22	
Vascular (Other Aneurysmal Diseases (e.g., Popliteal, Carotid, Pseudo-aneurysm))*		74.22	
Urology (Male Infertility)*		73.44	
Hepatobiliary (Choledochal Cyst)*		71.88	
Hepatobiliary (Sclerosing Cholangitis)*		71.88	
Pancreas (Periampullary Neoplasm)*		71.88	
Small Intestine (Radiation Enteritis)*		71.88	
Pancreas (Pancreatic Fistula)*		71.09	
Stomach and Duodenum (Gastric Polyps)*		71.09	
Vascular (Hemodialysis Access)*		71.09	
Hepatobiliary (Ampullary Stenosis/Sphincter of Oddi Dysfunction)*		70.31	
Liver (Liver Congenital Diseases)*		70.31	
Liver (Budd-Chiari Syndrome)*		70.31	
Hepatobiliary (Gallbladder Polyps)*		69.53	
Spleen (Splenic Tumors)*		69.53	
Abdomen - General / Hernia (Management of Retroperitoneal Tumors)*		68.75	
Breast (Breast Reconstruction)*		64.84	
Others (Transplantation)*		63.28	
Pancreas (Management of Pancreatic Congenital Anomalies (e.g., pancreas divisum))*		60.94	

Modified Delphi round 2 results

In the second round, 99.6% of content areas reached 75% consensus among 31 experts (Appendix 5). The highest prioritization was given to basic principles like the management of wounds and wound healing, fluids and electrolytes in surgery, physiological response to trauma, sepsis and infection, preoperative preparation, antibiotics use in surgery, ethics and surgery, surgical quality and safety, immediate postoperative care, and imaging in surgery. Abdominal conditions including appendicitis, typhoid enteritis, typhoid ileal perforation, small intestinal obstruction, gastric outlet obstruction, peptic ulcer diseases, and upper gastrointestinal bleeding were also high-priority areas. Pediatric conditions like intussusception, approach to bilious vomiting in the newborn, and pediatric fluid and electrolyte management were highly prioritized. Highest ranking anorectal conditions included hemorrhoids and anal fissures. Breast pathologies including cancer and infections, urologic conditions including acute urinary retention and hematuria, and orthopedic principles of fracture management were also ranked high priority for medical students in Rwanda (Appendix 5). Highest and lowest priority surgical topics are shown in Table 2. Overall, the consistency and average agreement between panel experts was strong. ICC was 0.856 (95% CI: 0.83-0.87), using Shrout and Fleiss 1979 Convention 3,k for 247 subjects and 31 judges/measurements, consistency, and a two-way mixed-effects model. The total Cronbach's Alpha for round 2 was very strong (0.985, 95% CI: 0.976-0.991) and higher than round 1, demonstrating strong reliability.

**Table 2:** Highest and Lowest Priority Areas of Surgical Teaching in Medical Schools to Prepare for General Practitioners for Surgical Care in Rwanda ^a^Aggregate prioritization score is the sum of assigned scores (exclude=1, not important=2, somewhat important=3, important= 4). ^b^Experts in agreement is the number of experts that rate the item as 'important' (4) or 'somewhat important' (3) ^c^Item-level content validity index (I-CVI) is the proportion of content experts giving item a rating of 'important' (4) or 'somewhat important' (3) ^d^Universal Agreement (UA) assigns 1 to the items that achieve 100% experts in agreement, and 0 if not all experts rate at 'important' (4) or 'somewhat important' (3) Scale-level content validity index based on the average method (S-CVI/Ave) using proportion relevance) = 0.935. Any S-CVI/Ave ≥ 0.9 implies excellent content validity. *Excluded. Intra-class correlation (ICC) estimates and their 95% confidence intervals for prioritization were calculated using JASP statistical package based on a mean-rating (k = 31) of multiple raters (247 subjects and 31 judges/measurements), consistency, 2-way mixed-effects model. Shrout and Fleiss (1979) Convention ICC 3,k point estimate 0.856 (95% CI: 0.83–0.87). Cronbach’s alpha = 0.985 (95% CI: 0.976–0.991).

Topic Areas	Aggregate Prioritization Score^a^	Experts in Agreement^b^	Universal Agreement (UA)^c^	Item-Level Content Validity index (I-CVI)^d^
Highest Priority Areas				
Surgery Overview (Wounds and Wound Healing)	100	31	1	1.00
Surgery Overview (Fluids and Electrolytes in Surgery)	100	31	1	1.00
Large Bowel([Appendicitis)	100	31	1	1.00
Pediatric (Intussusception)	99.19354839	31	1	1.00
Surgery Overview (Physiologic Response to Trauma)	99.19354839	31	1	1.00
Surgery Overview (Sepsis and Infection)	99.19354839	31	1	1.00
Surgery Overview (Preoperative Preparation)	99.19354839	31	1	1.00
Surgery Overview (Antibiotics Use in Surgery)	99.19354839	31	1	1.00
Surgery Overview (Ethics and Surgery)	99.19354839	31	1	1.00
Small Intestine (Typhoid Enteritis/Typhoid Ileal Perforation)	99.19354839	31	1	1.00
Small Intestine (Small Intestinal Obstruction)	99.19354839	31	1	1.00
Anorectal (Hemorrhoids)	99.19354839	31	1	1.00
Breast (Breast Infection)	99.19354839	31	1	1.00
Urology (Acute Urinary Retention)	99.19354839	31	1	1.00
Urology (Testicular Torsion)	99.19354839	31	1	1.00
Surgery Overview (Surgical Quality and Safety)	98.38709677	31	1	1.00
Surgery Overview (Immediate Postoperative Care)	98.38709677	31	1	1.00
Stomach and Duodenum (Gastric Outlet Obstruction)	98.38709677	31	1	1.00
Stomach and Duodenum (Peptic Ulcer)	98.38709677	31	1	1.00
Stomach and Duodenum (Upper Gastrointestinal Bleeding)	98.38709677	31	1	1.00
Anorectal (Anal Fissure)	98.38709677	31	1	1.00
Breast (Breast Cancer)	98.38709677	31	1	1.00
Pediatric (Fluid and Electrolyte Management)	98.38709677	31	1	1.00
Urology (Hematuria)	98.38709677	31	1	1.00
Orthopedic Surgery (Principles of Fracture Management)	98.38709677	31	1	1.00
Lowest Priority Areas				
Hepatobiliary (Cholangiography)	79.83870968	26	0	0.84
Stomach and Duodenum (Gastric Lymphoma)	79.83870968	26	0	0.84
Large Bowel (Appendiceal Neoplasm)	79.83870968	26	0	0.84
Thoracic (Mediastinal Tumors )	79.83870968	25	0	0.81
Orthopedic Surgery (Metabolic Bone Diseases)	79.83870968	28	0	0.90
Others (The Hidden Curriculum (Non-Explicit Learning from the Surgical Environment)	79.83870968	28	0	0.90
Stomach and Duodenum (Obesity)	79.03225806	25	0	0.81
Endocrine (Multiple Endocrine Neoplasia)	79.03225806	25	0	0.81
Plastic Surgery (Flaps)	79.03225806	25	0	0.81
Others (Introduction to Laparoscopic Surgery)	79.03225806	26	0	0.84
Hepatobiliary (Biliary Duct Tumors)	78.22580645	25	0	0.81
Large Bowel (Colon Carcinoid)	78.22580645	27	0	0.87
Neurosurgery (Brain Tumors)	78.22580645	26	0	0.84
Hepatobiliary (Biliary Duct Injury)	77.41935484	22	0	0.71
Hepatobiliary (Gallbladder Cancer)	76.61290323	23	0	0.74
Liver (Surgery in the Cirrhotic Patient)	76.61290323	24	0	0.77
Liver (Hepatic Biopsy)	75.80645161	25	0	0.81
Hepatobiliary (Biliary Surgery)*	71.77419355	23	0	0.74

Consensus conference

All 246 topics from the final round were presented, discussed, and verbally accepted by all 40 participants in open forum discussions during the consensus conference. Prioritization was accepted with no “stand asides” or blocking of consensus. The conference also came to a consensus to repeat the process every five years to maintain current relevance.

## Discussion

Using this multistage consensus process, we primarily present a list of prioritized surgery education topics that are relevant to the Rwandan context. These content areas have been adopted by medical schools in Rwanda. These will help focus curriculum development and form the basis for discussions on both Entrustable Professional Activities (EPAs) for new medical schools and curriculum reform for established institutions [31]. EPAs are an emerging pedagogical tool gaining traction in the teaching and training of medical students and post-graduates globally to ensure competence and trustworthiness in discrete professional tasks [32]. Secondarily, based specifically on the results of the first round, we present a starting point for similar discussions for other SSA countries. While every context is inherently different, our initial literature review and the multi-country first round of consensus building can be adapted for discussion in other countries in SSA. This can serve as a springboard from which to begin answering questions about what should be taught in other parts of East Africa, and other African sub-regions.

The argument has not yet been made for a unified undergraduate curriculum across all of Africa. Unlike some recognized close-knit regions with similar health systems and medical qualifying examinations [24], SSA has a diversity of surgical pathology specific to geographical, cultural, and social contexts. For this reason, one unified curriculum for all of SSA may be inappropriate. However, the argument should be made for contextual undergraduate surgical curricula in various parts of SSA. Surgical training curricula for the Global South cannot simply be adopted with little alteration from institutions from the Global North. Various factors, including country-specific practice expectations, surgeon, anesthetist, and obstetrician density and distribution, burden and variety of surgical disease, the extent of pathology at presentation, resources for diagnosis and treatment, and the stage of training at which graduates begin direct care of surgical patients, vary [33,34]. Therefore, surgical educators should strive to contextualize and prioritize locally relevant epistemology, medical illustrations, textbooks, manikins, and simulation materials bearing in mind indigenous knowledge, local research, and relatable skin tones.

The most highly prioritized content from this study included universal principles and foundations of general surgical care, management of acute emergencies, and a wide range of common surgical pathologies. Hepatobiliary surgical pathologies and training on the management of complicated surgical patients (surgery in the cirrhotic patient) were largely perceived to be out of scope for the non-specialist general practitioner. Rarer pathologies in the specific context like obesity, colon carcinoid, pilonidal disease, and metabolic bone diseases were also de-emphasized. Indeed, the mantra that “common things occur commonly” is reflected in the consensus. Of note is the fact that although an introduction to laparoscopic surgery attained consensus, it was in the lowest fifth percentile for this curriculum focused on junior medical student clerks.

We also illustrate the role of non-specialist physician surgical providers in helping define surgical content [35]. General practitioners who had lived experience in rural district hospitals were involved in each round and the consensus conference. One recent national study shows that surgeons will often underestimate the importance of surgical topics and training for non-surgeons [36]. Thus, the inclusion of General Practitioners’ voices grounded the study in the needs of their daily practice. Other studies in developing or reviewing medical school curricula have emphasized the need for general practitioners’ input [35,37,38], as this is often the first phase of trainees' practice upon graduation.

Limitations

As part of our modification, more Rwandan practitioners and general practitioners were included in the second round and the consensus conference by design, and therefore, over 50% of participants were different from those involved in the first round. This has limited our ability to assess for internal consistency between rounds for this modified Delphi process. We, however, acknowledge that surgical health challenges are similar in sub-Saharan Africa, and results from the initial Delphi round that was more representative of sub-Saharan Africa will be useful for several similar African contexts. We also did not have adequate gender balance among the experts, as there are still currently very few female Rwandan surgical providers. Future processes should also consider a deliberate balance of experts by sub-specialty, as sub-specialty training may introduce bias in experts' prioritization preferences.

## Conclusions

This consensus process has helped define broad topic areas that are essential for the development of surgical curricula in Rwanda and SSA. This is particularly important as, in an attempt to address the local burden of disease and maximize human resources for health, most graduates from Rwandan medical schools will have 3-to-5-year obligations with the government to practice at rural district hospitals and need the prioritized surgical skills. In this era of globalization, this modified Delphi process has prioritized areas often neglected on global curricula which will also help heighten the global competitiveness of Rwandan medical graduates. These results do not represent a specific curriculum, but a prioritization of surgical teaching for the region. This prioritization raises the bar for competence required of local graduates, which makes them competitive in many parts of the global health market where such surgical skills are not required upon graduation from medical school but are reserved for residency. In addition to representing consensus on what should be taught to medical students with high priority, these results represent an attempt at defining the surgical scope of practice for the non-specialist general practitioner (medical school graduate) in context, which will be updated every five years. The prioritized results have been adopted by surgical training teams at both the University of Rwanda and the University of Global Health Equity and will be implemented in curriculum mapping exercises, the design of EPAs, competency-based medical education, teaching and learning interventions, and simulation-based learning. The result of this consensus process is also useful for surgical education in similar contexts. Priorities for surgical teaching in the context have been clarified, and these should inform the hierarchy of teaching emphasis and the focus of academic resource- and partnership-building.

## Appendices

Appendix 1: Drafting of initial surgery topic list and defining of broad categories

Important grey literature and web-based sources from which our surgery topic list was developed include the following (Table 3):

**Table 3:** (Appendix 1) Important Grey Literature and Web-Based Sources From Which Our Surgery Topic List Was Developed

Documents	Source
1. The Rwanda Medical and Dental Council, Core Undergraduate MBBS curriculum	Institution Copy
2. Addis Ababa University/Ethiopian Medical School Surgery Curriculum.	Institution Copy
3. Jos University Teaching Hospital surgical curriculum	Institution Copy
4. All India Institute of Medical Sciences (AIIMS) MBBS syllabus.	https://www.aiims.edu/aiims/academic/aiims-syllabus/Syllabus%20-%20MBBS.pdf
5. UpToDate	https://www.uptodate.com/ [Accessed 31/05/2021]
6. Singh P. Important Topics in Surgery for NEET-PG	https://medical.prepladder.com/blog/1197-important-topics-in-surgery-for-neet-pg-by-dr-pritesh-singh.html
7. ACS/ASC Medical Student Core Curriculum	https://www.facs.org/education/program/core-curriculum [Accessed 31/05/2021]
8. Tomorrow’s Doctors (GMC, 2009)	http://www.ub.edu/medicina_unitateducaciomedica/documentos/TomorrowsDoctors_2009.pdf
9. Medscape	https://www.medscape.com/medicalstudents/resource [Accessed 31/05/2021]
10. Surgical Council on Resident Education, SCORE Curriculum Outline for General Surgery Residency 2014-2015	http://www.absurgery.org/xfer/curriculumoutline2014-15.pdf [Accessed 31/05/2021]
11. National Undergraduate Curriculum in Surgery. Royal College of Surgeons England.	https://www.rcseng.ac.uk/careers-in-surgery/careers-support/national-undergraduate-curriculum-in-surgery/ [Accessed 31/05/2021]
12. Physician Assistant Education Association General Surgery End of Rotation™ EXAM TOPIC LIST	https://paeaonline.org/wp-content/uploads/imported-files/general-surgery-topic-list-20180705.pdf
13. Teach Me Surgery	available at https://teachmesurgery.com/
14. Royal College of Surgeons, Ireland curriculum	Institution Copy
15. University of Wisconsin Surgical curriculum	Institution Copy

Formal Search Strategy

Search string: (Surgery AND (Education, Medical, Undergraduate OR Clinical Clerkship)) AND Students, Medical AND Curriculum NOT (Obstetrics OR Gynecology OR Anatomy)

Timing: Time-limited from 2001 to 2021

Database: PubMed

Results: Search Yielded 1,932 results

Screening and topic extraction was done by one researcher at title and abstract levels. All surgical undergraduate topics suggested by the article were included if not already present in our curriculum synthesis from grey literature. Full text review of articles that suggested lists of medical school topics for surgery was done by one investigator (BA) and two research assistants. These lists were extracted from text, tables, or figures and integrated into the topic list.

Appendix 2: Included articles and topics derived from PubMed search

Data from search (PubMed) are shown in Table 4

**Table 4:** Covidence Data From Literature Search **Expert citation

Medical Student And Faculty Perceptions Of Undergraduate Surgical Training In The South African And Swedish Tertiary Institutions: A Cross-Sectional Survey.	Authors	Published Year	Published Month	Journal	Volume	Issue	Pages	Accession Number	DOI	Ref	Covidence #	Study	Notes
A comparison of unguided vs guided case-based instruction on the surgery clerkship.	Adamas-Rappaport WJ; Waer AL; Teeple MK; Benjamin MA; Glazer ES; Sozanski J; Poskus D; Ong E	2013	Nov-Dec	J Surg Educ	70	6	821-5		10.1016/j.jsurg.2012.06.014	24209662	#1541	Adamas-Rappaport 2013	Interpersonal and communication skills, Practice-based learning, Case-based instruction
Survey of teaching, research and conference experiences of paediatric surgical trainees in Nigeria.	Ademuyiwa AO; Ameh EA; Bode CO; Adejuyigbe O	2011	Jan-Apr	Afr J Paediatr Surg	8	1	04-Jul		10.4103/0189-6725.78659	21478577	#745	Ademuyiwa 2011	Urethral c ateterization, Sterile gloves donning
Medical education impact assessment: knowledge of final year medical students of Obafemi Awolowo University about male urethral catheterization.	Ademuyiwa AO; Eziyi AK	2010	Apr-Jun	Niger J Med	19	2	203-7		10.4314/njm.v19i2.56521	20642090	#1735	Ademuyiwa 2010	angiography setup, target vessel catheterization and intervention, stent placement, Lectures/tutorials, wardrounds, clinical demonstration in OPD
Introduction of Suturing Skills Acquisition into Undergraduate Surgical Education: Early Experience from Ile-Ife, Nigeria.	Aderounmu AA; Wuraola FO; Olasehinde O; Sowande OA; Adisa AO	2019	Jul-Dec	Niger J Surg	25	2	188-191		10.4103/njs.NJS_5_19	31579375	#287	Aderounmu 2019	Suturing skills, Handling of surgical instruments
A combined approach in prolonged COVID-19 pandemic to teach undergraduate surgery students-future primary care physicians.	Agarwal PK	2020	Nov	J Family Med Prim Care	9	11	5480-5483		10.4103/jfmpc.jfmpc_1129_20	33532382	#309	Agarwal 2020	Suturing, incision and drainage, chest tube insertion
Immediate Auditory Feedback is Superior to Other Types of Feedback for Basic Surgical Skills Acquisition.	Al Fayyadh MJ; Hassan RA; Tran ZK; Kempenich JW; Bunegin L; Dent DL; Willis RE	2017	Nov-Dec	J Surg Educ	74	6	e55-e61		10.1016/j.jsurg.2017.08.005	28865902	#1581	AlFayyadh 2017	Suturing skills, technical skills
A Video-Based Coaching Intervention to Improve Surgical Skill in Fourth-Year Medical Students.	Alameddine MB; Englesbe MJ; Waits SA	2018	Nov	J Surg Educ	75	6	1475-1479		10.1016/j.jsurg.2018.04.003	29699931	#1779	Alameddine 2018	Surgical skill, surgical education
Peer-led surgery education: A model for a surgery interest group.	Ali M; Babar Chauha SS; Noor A; Khan S; Enam SA	2021	Jan	J Pak Med Assoc	71(Suppl 1)	1	S112-S116			33582735	#912	Ali 2021	Skill development, personal development, mentorship, research
Attracting students to surgical careers: preclinical surgical experience.	Antiel RM; Thompson SM; Camp CL; Thompson GB; Farley DR	2012	May-Jun	J Surg Educ	69	3	301-5		10.1016/j.jsurg.2011.10.001	22483128	#1611	Antiel 2012	Knot tying, suturing, incision making, needle driver, holding pickups, laparascopic instruments, surgical simulators
Development and implementation of an introductory endovascular training course for medical students.	Aparajita R; Zayed MA; Casey K; Dayal R; Lee JT	2011	Nov	Ann Vasc Surg	25	8	1104-12		10.1016/j.avsg.2011.07.002	21945331	#9	Aparajita 2011	Mentored simulator sessions, didactic teachings
How to enhance interest in surgical specialties.	Avery DM	2008	Mar	Bull Am Coll Surg	93	3	38			18390230	#1417	Avery 2008	Suturing, knot tying, instrument identification, OR etiquette, basic laparoscopic skill
A novel approach to improve undergraduate surgical teaching.	Baker RC; Spence RA; Boohan M; Dorman A; Stevenson M; Kirk SJ; McGlade K	2015	Jan	Ulster Med J	84	1	30-Jun			25964701	#1828	Baker 2015	Acute abdomen, acute appendicitis, fluids and electrolytes, hernia, pre-operative assessment, gallstones, shock, colorectal carcinoma, abdominal aortic aneurysm, pain control, jaundice, post-operative complications, inflammatory bowel disease
Cognitive skills analysis, kinesiology, and mental imagery in the acquisition of surgical skills.	Bathalon S; Dorion D; Darveau S; Martin M	2005	Oct	J Otolaryngol	34	5	328-32		10.2310/7070.2005.34506	16181595	#941	Bathalon 2005	Kinesiology, mental imagery, ATLS
Trends in Retention and Decay of Basic Surgical Skills: Evidence from Addis Ababa University, Ethiopia: A Prospective Case-Control Cohort Study.	Bekele A; Wondimu S; Firdu N; Taye M; Tadesse A	2019	Jan	World J Surg	43	1	Sep-15		10.1007/s00268-018-4752-1	30097707	#781	Bekele 2019	Instrument identification, one-handed knot tying, suturing
Medical Students Teaching Medical Students Surgical Skills: The Benefits of Peer-Assisted Learning.	Bennett SR; Morris SR; Mirza S	2018	Nov	J Surg Educ	75	6	1471-1474		10.1016/j.jsurg.2018.03.011	29653841	#165	Bennett 2018	WHO surgical safety checklist, scrubbing, gowning/gloving, knot tying, interrupted sutures, continuous sutures, vertical mattress sutures, local anesthesia
Surgical boot camp for fourth-year medical students: Impact on objective skills and subjective confidence.	Bevilacqua LA; Simon J; Rutigliano D; Sorrento J; Wackett A; Chandran L; Talamini M; Docimo S Jr	2020	Feb	Surgery	167	2	298-301		10.1016/j.surg.2019.06.041	31427073	#132	Bevilacqua 2020	Patient hand-off, suturing, knot tying, central line placement, chest tube placement
Cutting too deep? Assessing the impact of a shorter surgery clerkship on students' clinical skills and knowledge.	Bhatia ND; Gillespie CC; Berger AJ; Hochberg MS; Ogilvie JB	2014	Feb	Am J Surg	207	2	209-12		10.1016/j.amjsurg.2013.07.035	24238603	#1407	Bhatia 2014	Blood pressure, orthostatic blood pressure, rectal exam, fecal occult blood test
A needs assessment study of undergraduate surgical education.	Birch DW; Mavis B	2006	Oct	Can J Surg	49	5	335-40			17152571	#1208	Birch 2006	Central venous line, thoracentesis
Procedural Skills of the Entrustable Professional Activities: Are Graduating US Medical Students Prepared to Perform Procedures in Residency?	Bruce AN; Kumar A; Malekzadeh S	2017	Jul-Aug	J Surg Educ	74	4	589-595		10.1016/j.jsurg.2017.01.002	28126380	#1538	Bruce 2017	Basic cardiopulmonary resuscitation, bag/mask ventilation, universal precaution, venipuncture, intravenous catheter placement, arterial puncture
Surgical undergraduate education in rural Australia.	Bruening MH; Maddern GJ	2002	Jul	Arch Surg	137	7	794-8		10.1001/archsurg.137.7.794	12093334	#1515	Bruening 2002	Intravenous access, rectal examination, proctoscopy, endotracheal intubation, urinary catheterization, sigmoidoscopy, gastrointestinal tract endoscopy, surgical scrub, tie surgical knots, administer local anaesthesia, close surgical wounds
Accelerated skills preparation and assessment for senior medical students entering surgical internship.	Brunt LM; Halpin VJ; Klingensmith ME; Tiemann D; Matthews BD; Spitler JA; Pierce RA	2008	May	J Am Coll Surg	206	5	897-904; discussion 904-7		10.1016/j.jamcollsurg.2007.12.018	18471719	#1831	Brunt 2008	Suturing, knot tying, chest tube insertion, laparoscopic skill, use of energy devices,
Assessment of EFAST training for final year medical students in emergency medicine clerkship.	Cevik AA; Noureldin A; El Zubeir M; Abu-Zidan FM	2018	Sep	Turk J Emerg Med	18	3	100-104		10.1016/j.tjem.2018.05.004	30191188	#577	Cevik 2018	Cardiopulmonary resuscitation, airway management, suturing, emergency case management
Students' Interest in Surgery Affects Laparoscopic Practicing Performance.	Cheng Luo C; Mao Wu S; Kuei Chien W; Sheng Huang C; Cheng Lin W; Chun Chang Y	2016	Jul-Sep	JSLS	20	3			10.4293/JSLS.2016.00039	27493472	#1712	ChengLuo 2016	Needle transferring, suture placement, knot-tying skills
Improvement in acute care surgery medical student education and clerkships: use of feedback and loop closure.	Cherry-Bukowiec JR; Machado-Aranda D; To K; Englesbe M; Ryszawa S; Napolitano LM	2015	Nov	J Surg Res	199	1	15-22		10.1016/j.jss.2015.05.062	26148827	#94	Cherry-Bukowiec 2015	Trauma, burn, surgical critical care, nontrauma emergency surgery
Plastic Surgery Undergraduate Training: How a Single Local Event Can Inspire and Educate Medical Students.	Daggett A	2016	Mar	Ann Plast Surg	76	3	364-5		10.1097/SAP.0000000000000736	26855036	#1584	Daggett 2016	Aesthetic suturing, local flap design, tendon repair
Surgical and procedural skills training at medical school - a national review.	Davis CR; Toll EC; Bates AS; Cole MD; Smith FC	2014		Int J Surg	12	8	877-82		10.1016/j.ijsu.2014.05.069	24909137	#1757	Davis 2014	Gowning, gloving, handling instruments, knot tying, suturing
Competence and confidence with basic procedural skills: the experience and opinions of fourth-year medical students at a single institution.	Dehmer JJ; Amos KD; Farrell TM; Meyer AA; Newton WP; Meyers MO	2013	May	Acad Med	88	5	682-7		10.1097/ACM.0b013e31828b0007	23524922	#1672	Dehmer 2013	Foley catheter insertion, nasogastric tube insertion, venipuncture, intravenous catheter insertion, arterial puncture, basic suturing, endotracheal intubation, lumbar puncture, thoracentesis
Senior medical student "Boot Camp": can result in increased self-confidence before starting surgery internships.	Esterl RM Jr; Henzi DL; Cohn SM	2006	Jul-Aug	Curr Surg	63	4	264-8		10.1016/j.cursur.2006.03.004	16843778	#2069	EsterlRMJr 2006	anatomic dissection, administrative skills, technical skills, patient management
Measuring medical students' experience with surgical problems and procedures.	Freeman RB; Rheinlander H	2001	Apr	Am J Surg	181	4	341-6		10.1016/s0002-9610(01)00572-4	11438269	#1511	Freeman 2001	post op fever, shock, hemorrhage, breast mass, groin mass, scrotal mass, dysphagia, heartburn, cough, hemoptysis, acute abdominal pain, appendicitis, gallbladder disease, jaundice, intestinal obstruction, blood in stool, anorectal disease, carotid bruit, pulsating abdominal mass, intermittent claudication, varicose veins, acute leg pain, acute leg swelling, coronary artery disease, enlarged prostate, flank pain, extremity trauma, back pain, neck mass, sore throat, ear ache, pigmented skin lesion, thermal injury, blunt trauma, penetrating trauma, pain management, CPR, right progress notes, write post operative orders, phlebotomy for blood sampling, insert peripheral Ivy line, insert central venous line, insert Swanz Ghaz line, arterial puncture, lumbar puncture, paracentesis, thoracocentesis, thoracostomy, bladder catheterization, surgical scrub for operating room, prepare sterile surgical field, bandage wound, dress surgical wound, suture surgical wound, remove sutures, describe and irrigate contaminated wound, local anesthesia, incise and drain simple abscess, apply splints, apply simple casts, write post operative orders, write progress notes, cardio pulmonary resuscitation, medication injections.
Blended learning in surgery using the Inmedea Simulator.	Funke K; Bonrath E; Mardin WA; Becker JC; Haier J; Senninger N; Vowinkel T; Hoelzen JP; Mees ST	2013	Feb	Langenbecks Arch Surg	398	2	335-40		10.1007/s00423-012-0987-8	22878596	#1604	Funke 2013	Incarcerated inguinal hernia, Acute cholecystitis with cholecystolithiasis, Sigmoid diverticulitis, Esophageal cancer, Acute mesenteric infarction, Acute cholecystitis with choledocholithiasis Exercise, Thromboembolic disease of femoral artery, Abdominal aortic aneurysm, Cholangiocarcinoma with jaundice
Medical student electives in general surgical subspecialties.	Gabram SG; Hoenig J; Creech S; Minks KD; Gamelli RL	2004	Sep	Am J Surg	188	3	246-9		10.1016/j.amjsurg.2004.03.010	15450828	#428	Gabram 2004	burn, trauma, and peripheral vascular
[It's all in Your Head! Influence of Mental Training on the Acquisition of Practical Skills in Surgical Training].	Germanyuk A; Sterz J; Stefanescu C; Vo√ü SH; R√ºsseler M	2019	Dec	Zentralbl Chir	144	6	597-605		10.1055/a-1031-9711	31826294	#504	Germanyuk 2019	applying local anaesthetic, sterile covering and preparation of wound, performing simple interrupted suture
Surgical Skills Workshops Should Be a Part of the United Kingdom Undergraduate Medical Curriculum.	Hakim MA; Dominguez ED; Priest S; Lee KS; Mardanpour A; Tandle S; Al-Khalil M; Slade G; Gujral S	2019	May	Cureus	11	5	e4642		10.7759/cureus.4642	31312568	#359	Hakim 2019	Suturing, Dynamic Hip Screw, Laparoscopic Simulation, Burr Hole Drilling, Trauma Scenarios, Tendon Repair, Aortic Re-Anastomosis, Tracheostomy
Utilizing Surgical Bootcamps to Teach Core Entrustable Professional Activities to Senior Medical Students.	Heimroth J; Jones VM; Howard JD Jr; Sutton ERH	2018	Jun	Am Surg	84	6	783-788			29981602	#1618	Heimroth 2018	airway management, venous access, basic surgical technique, patient care, radiol- ogy, and obstetric, orthopedic, and plastic surgery emergencies, Death on the Wards
Utilization of a non-preserved cadaver to address deficiencies in technical skills during the third year of medical school: a cadaver model for teaching technical skills.	Kaplan SJ; Carroll JT; Nematollahi S; Chuu A; Adamas-Rappaport W; Ong E	2013	May	World J Surg	37	5	953-5		10.1007/s00268-013-1905-0	23354919	#1229	Kaplan 2013	basic suturing, intubation, cricothyrotomy, chest tube placement, thoracentesis, venous access, central line, radial arterial line placement
The use of theatre in medical education in the emergency cases school: an appealing and widely accessible way of learning.	Keskinis C; Bafitis V; Karailidou P; Pagonidou C; Pantelidis P; Rampotas A; Sideris M; Tsoulfas G; Stakos D	2017	Jun	Perspect Med Educ	6	3	199-204		10.1007/s40037-017-0350-4	28405926	#610	Keskinis 2017	Acute pancreatitis Myocardial infarction Acute lower limb ischaemia Subarachnoid haemorrhage Ureterolithiasis Duodenal ulcer Cerebrovascular accident Under tension pneumothorax Cauda equina syndrome, Peptic ulcer disease Myocardial infarction Cholecystitis Gastric cancer Oesophagitis Acute aortic dissection Acute pericarditis Pulmonary embolism Infective endocarditis Deep venous thrombosis Vasculitis Vasospasm Peripheral vascular injury Subdural/epidural/endocranial haematoma Migraine Meningitis, encephalitis Transient ischaemic attack Obstructive blood clot in the ureter Renal vein thrombosis Biliary colic Appendicitis Ureter carcinoma Acute cholangitis Acute pancreatitis Acute gastritis Inflammatory bowel disease Gastroesophageal reflux disease Seizure Systemic infection Brain tumour Metabolic disorders Vertigo Acute respiratory distress syndrome Pneumonia Chronic obstructive pulmonary disease Pulmonary embolism Myocardial infarction Multiple sclerosis Neoplasms of the spinal cord Diabetic neuropathy
The Sex Difference in Basic Surgical Skills Learning: A Comparative Study.	Lou Z; Yan FH; Zhao ZQ; Zhang W; Shui XQ; Liu J; Zhuo DL; Li L; Yu ED	2016	Sep-Oct	J Surg Educ	73	5	902-5		10.1016/j.jsurg.2016.04.002	27184180	#1824	Lou 2016	knot tying, basic suture I, basic suture II, sterile technique, preoperative preparation, phlebotomy, debridement, laparotomy, cecectomy, small bowel resection with hand-sewn anastomosis
Undergraduate basic surgical skills education: impact on attitudes to a career in surgery and surgical skills acquisition.	McAnena PF; O'Halloran N; Moloney BM; Courtney D; Waldron RM; Flaherty G; Kerin MJ	2018	May	Ir J Med Sci	187	2	479-484		10.1007/s11845-017-1696-7	29043542	#1670	McAnena 2018	basic interrupted suture, surgical scrubbing
Implementation of an E-Learning Academic Elective for Hands-On Basic Surgical Skills to Supplement Medical School Surgical Education.	McGann KC; Melnyk R; Saba P; Joseph J; Glocker RJ; Ghazi A	2020	Dec	J Surg Educ					10.1016/j.jsurg.2020.11.014	33309226	#267	McGann 2020	surgical instrument identification, knot tying, suturing
Teaching technical skills to medical students during a surgery clerkship: results of a small group curriculum.	Meyers MO; Meyer AA; Stewart RD; Dreesen EB; Barrick J; Lange PA; Farrell TM	2011	Apr	J Surg Res	166	2	171-5		10.1016/j.jss.2010.05.019	20828751	#54	Meyers 2011	Foley catheter placement, NG tube insertion/removal, IV placement, arterial puncture
Can medical students achieve skills proficiency through simulation training?	Naylor RA; Hollett LA; Valentine RJ; Mitchell IC; Bowling MW; Ma AM; Dineen SP; Bruns BR; Scott DJ	2009	Aug	Am J Surg	198	2	277-82		10.1016/j.amjsurg.2008.11.036	19362285	#1552	Naylor 2009	bladder catheterization, breast examination, knot-tying
Integrating a Novel Global Surgery and Health Inequity Course to the Surgical Clerkship.	Padmanaban V; Fallah P; Jayaraman S; Peck GL; Sifri Z	2020	Sep-Oct	J Surg Educ	77	5	1106-1112		10.1016/j.jsurg.2020.03.014	32534939	#229	Padmanaban 2020	surgical abdominal examination, urethral catheterization, rectal-digital examination, handling of central venous catheters
DOPS (Direct Observation of Procedural Skills) in undergraduate skills-lab: Does it work? Analysis of skills-performance and curricular side effects.	Profanter C; Perathoner A	2015		GMS Z Med Ausbild	32	4	Doc45		10.3205/zma000987	26483858	#882	Profanter 2015	Drain care/removal, staple removal, Steri-Strip application
An electronic portfolio for quantitative assessment of surgical skills in undergraduate medical education.		Sánchez Gómez, S., Ostos, E.M.C., Solano, J.M.M.	2013	May	BMC Med Educ	13		65	10.1186/1472-6920-13-65	23642100	#1555	Sánchez Gómez	Otorhinolaryngology outpatient office Apparatus, instruments and pharmacology. Hand washing Otorhinolaryngology medical record Complementary studies Preoperative evaluation Operated patients evaluation Post-surgery protocols: pharmacology, cures, laboratory studies Patient information and Informed Consent Otorhinolaryngology documents Hospitalization Otorhinolaryngology hospitalization ward Otorhinolaryngology medical record Complementary studies Preoperative evaluation Patient preparation for surgery. Antibiotic prophylaxis. Hematologic prophylaxis Apparatus, instruments Emergencies Surgical patients management Wound care Administration of drugs Drains and drain management Surgery ward security protocols Diets, fluid therapy Operating room Otorhinolaryngology operating room Professional behavior in an otorhinolaryngology operating room Sterility, surgical hand washing, gowns/cap/mask/gloves placement Patient preparation for surgery: position, personnel, and apparatus Apparatus, instruments and pharmaceuticals Surgical site preparation: sterile drapes, antiseptic techniques Sutures: materials, types, mechanisms, and procedures Operating room security protocols Operating room documents Surgical techniques: fundamentals, indications, proceduresSuturing/knot tying, urinary catheter, sterile prep/drape, IV placement, informed consent, electrosurgical use	
Medical Student And Faculty Perceptions Of Undergraduate Surgical Training In The South African And Swedish Tertiary Institutions: A Cross-Sectional Survey.	Scott AJ; Drevin G; Pavloviƒá L; Nilsson M; Krige JE; Jonas E	2019		Adv Med Educ Pract	10		855-866		10.2147/AMEP.S216027	31686944	#346	Scott 2019	Basic surgical anatomy, Proper surgical history, Proper surgical history, Ability to present patients,
Use of a hybrid-abdominal wound simulated patient in the ACS/ASE medical student simulation skills curriculum.	Shariff FU; Deladisma AM; Menard JW; Shewokis PA; Lind DS	2019	Feb	Am J Surg	217	2	250-255		10.1016/j.amjsurg.2018.07.039	30078670	#40	Shariff 2019	Drains. Communication skills and patient interaction, Staple removal and Steri-Strip application
Trauma leagues: an alternative way to teach trauma surgery to medical students.	Simões RL; Bermudes FA; Andrade HS; Barcelos FM; Rossoni BP; Miguel GP; Fagundes CA; Fraga GP	2014	Jul-Aug	Rev Col Bras Cir	41	4	297-302		10.1590/0100-699120140040013	25295993	#902	Simões 2014	Pre-hospital care Airways and cervical protection Breathing Circulation Neurology Exposure and hypothermia control Advanced trauma procedures Trauma imaging
Outcomes of a proficiency-based skills curriculum at the beginning of the fourth year for senior medical students entering surgery.	Wade TJ; Lorbeer K; Awad MM; Woodhouse J; DeClue A; Brunt LM	2015	Oct	Surgery	158	4	962-9; discussion 969-71		10.1016/j.surg.2015.07.005	26283204	#1752	Wade 2015	Suture and knot tying tasks Simple interrupted suture Subcuticular suture One hand tie Two hand tie Tie on pass Laparoscopic skills Camera navigation Peg transfer Bean drop* Pattern cutting* Sterile prep and drape Informed consent Peripheral IV insertion Urinary catheter placement Basic electrosurgical use* On-call problem management Hypotension Tachycardia Altered mental status Dyspnea
**Baqal OJ, Soheib M, Saadallah AA. Peer-Led Surgical Safety Learning Among Medical Students Using a Novel Story-Based Approach. Cureus. 2020 Sep 4;12(9):e10242. doi: 10.7759/cureus.10242. PMID: 33042682; PMCID: PMC7537487.		2020	Sep	Cureus					doi: 10.7759/cureus.10242.			Baqual 2020	The basics of surgical safety, WHO’s Surgical Safety Checklist, Understanding systems and impact of complexity on patient care, Human factors, Being a team player and showing leadership, Managing fatigue and stress, Involving patients and carers as partners in healthcare, Communicating honestly with patients after an adverse event (open disclosure)

Appendix 3: Initial surgical topics and domains

The list of all topics and domains is shown in Table 5.

**Table 5:** (Appendix 3) Initial Surgery Topics and Domains

	Domains	Topics and Subject Areas
1.	Introduction to Surgery	
		History of Surgery
		Introduction to Global Surgery
		Non technical skills for surgery
		Surgical quality and safety
		Neuroendocrine response to trauma
		Wounds and wound healing
		Classification and management of wounds
		Sepsis and infection
		Fluid and electrolytes in surgery
		Nutritional optimisation
		Preoperative preparation
		Communication in surgery
		Consent for surgery
		Antibiotics use in Surgery
		Ethics and surgery
		Imaging in surgery
2.	Abdomen	
2a.	General Abdomen	Management of the Acute Abdomen
		Differential diagnosis of a right iliac fossa mass
		Management of abdominal wall tumors
		Management of retroperitoneal tumors
2b.	Herniae	Diaphragmatic hernia
		Inguinal hernia
		Femoral hernia
		Incisional hernia
2c.	Hepatobiliary	Ampullary stenosis/sphincter of Oddi dysfunction
		BIliary duct injury
		Biliary duct tumors
		Biliary disease diagnosis
		Biliary pancreatitis
		Biliary surgery
		Obstructive jaundice
		Cholangitis
		Cholangiography
		Cholecystitis
		Choledochal cyst
		Choledocholithiasis
		Gallbladder cancer
		Gallbladder polyps
		Gallstone ileus
		Sclerosing cholangitis
2d.	Liver	Budd-Chiari syndrome
		Portal hypertension
		Hepatic biopsy
		Hepatic mass
		Hepatic neoplasms
		Hepatic injury
		Liver abscesses
		Liver hydatid diseases
		Liver congenital diseases
2e.	Pancreas	Management of pancreatic congenital anomalies (e.g pancreas divisum)
		Pancreatic neoplasm
		Pancreatic fistula
		Pancreatic pseudocyst
		Chronic pancreatitis
		Acute Pancreatitis
		Periampullary neoplasm
2f.	Spleen	Management of splenic trauma
		Post splenectomy syndrome
		Splenic Abscess
		Splenomegaly
		Splenectomy
		Splenic tumors
2g.	Stomach and duodenum	Gastric cancer
		Gastric GIST
		Gastric lymphoma
		Gastric polyps
		Gastric volvulus
		Obesity
		Peptic ulcer
		Postgastrectomy syndromes
		Stress gastritis
		Upper gastrointestinal bleeding
2h.	Large Bowel	Appendicitis
		Appendiceal neoplasm
		Colon cancer
		Colon carcinoid
		Colon polyps
		Diverticular disease
		Colovesical fistulas
		Colostomy
		Functional colonic disorders
		Large bowel obstruction
		Volvulus
		Malignant bowel obstruction
		Lower gastrointestinal bleeding
		Ulcerative colitis
2i.	Small intestine	Gastro-intestinal fistula (Enterocutaneous and entero-atmospheric)
		Typhoid enteritis/Typhoid ileal perforation
		Meckel's diverticulum
		Mesenteric Ischaemia
		Paralytic ileus
		Radiation enteritis
		Small intestinal carcinoid tumour
		Small intestinal lymphoma
		Small intestinal obstruction
		Gastrointestinal stromal tumour
2j.	Anorectal	Anal cancer
		Anal fissure
		Anorectal abscess and fistulae
		Faecal incontinence
		Haemorrhoids
		Rectal cancer
		Rectal prolapse
3.	Oesophagus	
		Dysphagia
		Barrett's oesophagus
		Benign esophageal tumor
		Oesophageal cancer
		Esophageal stricture
		Esophageal diverticula
		Esophageal foreign body
		Esophageal motility disorders
		Esophageal perforation
		Gastroesophageal reflux
		Hiatal hernia
4.	Thoracic surgery	
		Thoracic trauma
		Surgical Lung Infections (Lung abscess, bronchiectasis, empyema)
		Lung TB
		Benign lung tumours
		Lung cancer
		Mediastinal tumours
5.	Vascular	
		abdominal aortic aneurysm
		Arterial access
		Central venous access
		Vascular trauma
		Varicose veins
		Hemodialysis access
6.	Breast	
		Benign breast disease
		Breast cancer
		Breast carcinoma in-situ
		Paget's disease
		Breast sarcoma
		Gynaecomastia
		Phyllodes tumor
		Breast infection
7.	Endocrine	
		Goitres
		Multiple endocrine neoplasia
		Adrenal tumours
		Hyperparathyroidism
		Parathyroid tumours
		Pheochromocytoma
8.	Urology	
		Benign Prostate Hypertrophy
		Cancer of the Prostate
		Prostatitis
		Acute urinary retention
		Bladder tumours
		Haematuria
		Urethral stricture
		Posterior urethral valves
		Testicular tumour
		Renal tumour
		Undescended testes
		Carcinoma of the penis
		Penile fracture
		Hypospadias
		Epispadias
		Male infertility
		Urolithiasis
		Renal trauma
		Urethral stricture
		Renal cysts
		Urogenital fistula
		Urologic trauma (including iatrogenic)
		Varicocoele
		Incontinence
9.	Ear Nose and Throat surgery	
		Epistaxis
		Neck mass
		Sialolithiasis
		Tonsillitis; Adenoiditis
		Oropharyngeal cancer
		Neck trauma
10.	Plastic surgery	
		Cleft lip and palate
		Skin tumors
		Burns
		Skin graft and flaps
11.	Orthopaedic Surgery	
		Leg ulcers
		The Painful/the Paralysed Limb
		Volkmann Ischaemic Contracture
		Carpal tunnel syndrome
		Wrist drop development and management
		the swollen/painful knee
		Cord compression syndromes
		Principles of fracture management
		Bone and joint infections
		Bone tumours
		Metabolic bone diseases
		Congenital deformities
		Amputations and disarticulations
		Tropical leg ulcers
		Diabetic foot ulcers
12.	Others	
		Patient positioning
		Surgery in the elderly
		The difficult patient encounter
		Reflexive Writing
		Surgical Risk Assessment
		Spirituality and surgery
		Acute and chronic pain management
		The hidden curriculum
		Introduction to laparoscopic surgery
		HIV/AIDs and the Surgical Patient
		Surgical audit and Research
		Day care surgery
		Transplantation
		Acute airway obstruction in adults and children

Appendix 4: Modified Delphi round 1 prioritization

**Table 6:** (Appendix 4) Modified Delphi Round 1 Prioritization

Surgical Thematic Areas [Topic]	Aggregate Prioritization Score	Disposition
Surgery Overview [Sepsis and Infection]	100	Include
Surgery Overview [Antibiotics Use in Surgery]	100	Include
Abdomen - General / Hernia [Management of Acute Abdomen]	100	Include
Abdomen - General / Hernia [Abdominal Pain]	100	Include
Spleen [Management of Splenic Trauma]	100	Include
Stomach and Duodenum [Upper Gastrointestinal Bleeding]	100	Include
Large Bowel [Appendicitis]	100	Include
Large Bowel [Large Bowel Obstruction]	100	Include
Anorectal [Anorectal Abscess and Fistulae]	100	Include
Plastic Surgery [Burns]	100	Include
Orthopedic Surgery [Principles of Fracture Management]	100	Include
Orthopedic Surgery [Swollen/Painful Knee]	100	Include
Surgery Overview [Fluids and Electrolytes in Surgery]	99.2	Include
Stomach and Duodenum [Peptic Ulcer]	99.2	Include
Large Bowel [Lower Gastrointestinal Bleeding]	99.2	Include
Anorectal [Hemorrhoids]	99.2	Include
Breast [Breast Cancer]	99.2	Include
Urology [Acute Urinary Retention ]	99.2	Include
Urology [Hematuria]	99.2	Include
Orthopedic Surgery [Approach to Upper Extremity Fractures and Dislocations]	99.2	Include
Orthopedic Surgery [Approach to Lower Extremity Fractures and Dislocations]	99.2	Include
Orthopedic Surgery [Extremity Compartment Syndrome]	99.2	Include
Surgery Overview [Wounds and Wound Healing]	98.4	Include
Surgery Overview [Classification and Management of Wounds]	98.4	Include
Abdomen - General / Hernia [Inguinal Hernia]	98.4	Include
Abdomen - General / Hernia [Approach to Blunt and Penetrating Abdominal Trauma]	98.4	Include
Hepatobiliary [Cholecystitis]	98.4	Include
Small Intestine [Typhoid Enteritis/Typhoid Ileal Perforation]	98.4	Include
Small Intestine [Small Intestinal Obstruction]	98.4	Include
Large Bowel [Colostomy]	98.4	Include
Large Bowel [Volvulus]	98.4	Include
Anorectal [Anal Fissure]	98.4	Include
Anorectal [Rectal Cancer]	98.4	Include
Thoracic [Thoracic Trauma]	98.4	Include
Breast [Breast Infection]	98.4	Include
Breast [Breast Diagnostics]	98.4	Include
Pediatric [Inguinal Hernia]	98.4	Include
Pediatric [Intussusception]	98.4	Include
Urology [Testicular Torsion]	98.4	Include
Others [Responding to Common Peri-operative Problems (e.g., fever, tachycardia, chest pain, bleeding)]	98.4	Include
Others [Feeding Access (Tubes, Lines, and Drains)]	98.4	Include
Surgery Overview [Preoperative Preparation]	97.7	Include
Surgery Overview [Consent for Surgery]	97.7	Include
Surgery Overview [Immediate Postoperative Care]	97.7	Include
Hepatobiliary [Obstructive Jaundice]	97.7	Include
Spleen [Splenomegaly]	97.7	Include
Stomach and Duodenum [Gastric Outlet Obstruction]	97.7	Include
Large Bowel [Colon and Rectal Cancer]	97.7	Include
Breast [Benign Breast Disease]	97.7	Include
Endocrine [Goiter]	97.7	Include
Pediatric [Umbilical Hernia]	97.7	Include
Pediatric [Circumcision]	97.7	Include
Urology [Benign Prostate Hypertrophy]	97.7	Include
Orthopedic Surgery [Diabetic Foot Ulcers]	97.7	Include
Neurosurgery [Head Trauma]	97.7	Include
Others [Acute Airway Obstruction in Adult and Pediatric Patients]	97.7	Include
Surgery Overview [Surgical Safety Checklist]	96.9	Include
Surgery Overview [Physiologic Response to Trauma]	96.9	Include
Surgery Overview [Ethics and Surgery]	96.9	Include
Liver [Liver Abscesses]	96.9	Include
Pancreas [Acute Pancreatitis]	96.9	Include
Esophagus [Dysphagia]	96.9	Include
Vascular [Deep Vein Thrombosis and Thromboembolic Disease]	96.9	Include
Breast [Evaluation of Breast Pain (Mastalgia, Mastitis)]	96.9	Include
Pediatric [Fluid and Electrolyte Management]	96.9	Include
Pediatric [Nutrition]	96.9	Include
Pediatric [Venous Access]	96.9	Include
Plastic Surgery [Approach to Hand Injuries]	96.9	Include
Orthopedic Surgery [Bone and Joint Infections]	96.9	Include
Surgery Overview [Surgical Quality and Safety]	96.1	Include
Stomach and Duodenum [Stress Gastritis]	96.1	Include
Thoracic [Surgical Lung Infections (Lung Abscess, Bronchiectasis, Empyema)]	96.1	Include
Endocrine [Thyroid Nodules]	96.1	Include
Pediatric [Gastroschisis and Omphalocele]	96.1	Include
Pediatric [Approach to Bilious Vomiting in the Newborn]	96.1	Include
Urology [Prostate Cancer]	96.1	Include
Orthopedic Surgery [Approach to Pelvic and Sacral Fractures]	96.1	Include
Orthopedic Surgery [Amputations and Disarticulations]	96.1	Include
Orthopedic Surgery [Tropical Leg Ulcers]	96.1	Include
Liver [Hepatic Injury]	95.3	Include
Large Bowel [Constipation]	95.3	Include
Esophagus [Esophageal Foreign Body]	95.3	Include
Esophagus [Gastro-esophageal Reflux]	95.3	Include
Endocrine [Thyroid Cancers]	95.3	Include
Pediatric [Pyloric Stenosis]	95.3	Include
Plastic Surgery [Skin Grafts]	95.3	Include
Plastic Surgery [Necrotizing Soft Tissue Infections]	95.3	Include
Abdomen - General / Hernia [Differential Diagnosis of a Right Iliac Fossa Mass]	94.5	Include
Abdomen - General / Hernia [Incisional Hernia]	94.5	Include
Hepatobiliary [Cholangitis]	94.5	Include
Hepatobiliary [Biliary Disease Evaluation]	94.5	Include
Stomach and Duodenum [Gastric Cancer]	94.5	Include
Large Bowel [Malignant Bowel Obstruction]	94.5	Include
Anorectal [Anal Cancer]	94.5	Include
Thoracic [Pulmonary and Pleural TB]	94.5	Include
Vascular [Central Venous Access]	94.5	Include
Vascular [Vascular Trauma]	94.5	Include
Breast [Breast Carcinoma In-Situ]	94.5	Include
Urology [Renal Trauma]	94.5	Include
Orthopedic Surgery [Approach to Spine Fractures]	94.5	Include
Others [Acute and Chronic Pain Management]	94.5	Include
Surgery Overview [Nutritional Optimization]	93.8	Include
Surgery Overview [Communication in Surgery]	93.8	Include
Surgery Overview [Imaging in Surgery]	93.8	Include
Surgery Overview [Outpatient Follow-up of Common Surgical Conditions and Long-Term Outcomes]	93.8	Include
Abdomen - General / Hernia [Femoral Hernia]	93.8	Include
Hepatobiliary [Choledocholithiasis]	93.8	Include
Spleen [Splenectomy]	93.8	Include
Small Intestine [Paralytic Ileus]	93.8	Include
Esophagus [Esophageal Cancer]	93.8	Include
Pediatric [Undescended Testes]	93.8	Include
Urology [Prostatitis and Epididymitis]	93.8	Include
Plastic Surgery [Approach to Hand Infections]	93.8	Include
Orthopedic Surgery [Tuberculosis of the Spine]	93.8	Include
Orthopedic Surgery [Cord Compression Syndromes]	93.8	Include
Neurosurgery [Hydrocephalus]	93.8	Include
Others [HIV/AIDS and the Surgical Patient]	93.8	Include
Surgery Overview [Non-technical Skills for Surgery]	93	Include
Anorectal [Rectal Prolapse]	93	Include
Endocrine [Graves Disease]	93	Include
Endocrine [Thyroiditis]	93	Include
Pediatric [Surgical Jaundice in the Newborn]	93	Include
Urology [Urethral Stricture]	93	Include
Orthopedic Surgery [Painful/Paralyzed Limb]	93	Include
Neurosurgery [Surgical CNS Infections]	93	Include
Surgery Overview [Introduction to Global Surgery]	92.2	Include
Liver [Portal Hypertension]	92.2	Include
Spleen [Splenic Abscess]	92.2	Include
Others [Difficult Patient Encounters]	92.2	Include
Others [Surgical Risk Assessment]	92.2	Include
Others [Surgical Audit and Research]	92.2	Include
Breast [Role of Community Health Workers in Prevention, Screening, and Community Sensitization of Breast Disease]	91.4	Include
Pediatric [Anorectal Malformations]	91.4	Include
Pediatric [Necrotizing Enterocolitis]	91.4	Include
Plastic Surgery [Skin Tumors]	91.4	Include
Surgery Overview [Introduction to Surgery/History of Surgery]	90.6	Include
Liver [Differential Diagnosis of Hepatic Masses]	90.6	Include
Spleen [Post Splenectomy Syndrome]	90.6	Include
Breast [Paget's Disease]	90.6	Include
Urology [Urolithiasis]	90.6	Include
Urology [Urologic Trauma (including iatrogenic)]	90.6	Include
Plastic Surgery [Keloids and Hypertrophic Scars]	90.6	Include
Neurosurgery [Acute Stroke Care]	90.6	Include
Hepatobiliary [Biliary Pancreatitis]	89.8	Include
Liver [Liver Hydatid Dieseases]	89.8	Include
Esophagus [Esophageal Stricture]	89.8	Include
Thoracic [Lung Cancer]	89.8	Include
Urology [Testicular Tumor]	89.8	Include
Orthopedic Surgery [Bone Tumors]	89.8	Include
Others [Patient Positioning]	89.8	Include
Abdomen - General / Hernia [Abdominal Compartment Syndrome]	89.1	Include
Liver [Parasitic Liver Diseases of Surgical Importance]	89.1	Include
Urology [Varicocele]	89.1	Include
Orthopedic Surgery [Volksman Ischemic Contracture]	89.1	Include
Anorectal [Fecal Incontinence]	88.3	Include
Esophagus [Esophageal Perforation]	88.3	Include
Pediatric [Gastrointestinal Tract Atresia and Stenosis]	88.3	Include
Others [Reflective Writing (medical student journaling to generate compassion for patients)]	88.3	Include
Small Intestine [Meckel's Diverticulum]	87.5	Include
Vascular [Peripheral Arterial Disease]	87.5	Include
Pediatric [Tracheo-esphogeal Congenital Anomalies]	87.5	Include
Orthopedic Surgery [Carpal Tunnel Syndrome]	87.5	Include
Neurosurgery [Neural Tube Defects]	87.5	Include
Others [Same-Day Surgery]	87.5	Include
Hepatobiliary [Parasitic Biliary Obstruction]	86.7	Include
Pancreas [Chronic Pancreatitis]	86.7	Include
Small Intestine [Gastrointestinal Fistula (Enterocutaneous and Enteroatmospheric)]	86.7	Include
Vascular [Lymphoedema]	86.7	Include
Pediatric [Pediatric Pleural Infections]	86.7	Include
Urology [Bladder Tumours]	86.7	Include
Urology [Renal Tumor]	86.7	Include
Vascular [Abdominal Aortic Aneurysm]	85.9	Include
Endocrine [Pheochromocytoma]	85.9	Include
Plastic Surgery [Cleft Lip and Palate]	85.9	Include
Small Intestine [Mesenteric Ischaemia]	85.2	Include
Large Bowel [Colon Polyps]	85.2	Include
Large Bowel [Diverticular Disease]	85.2	Include
Anorectal [Condyloma]	85.2	Include
Breast [Gynaecomastia]	85.2	Include
Others [Surgery in the Elderly]	85.2	Include
Pancreas [Pancreatic Neoplasm]	84.4	Include
Large Bowel [Inflammatory Bowel Disease (Ulcerative Colitis and Crohn's Disease)]	84.4	Include
Esophagus [Barrett's esophagus]	84.4	Include
Vascular [Arterial Access]	84.4	Include
Breast [Breast Sarcoma]	84.4	Include
Pediatric [Pediatric Neoplasms (e.g., Neuroblastoma, Nephroblastoma)]	84.4	Include
Pediatric [Hypospadias and Epispadias]	84.4	Include
Urology [Incontinence]	84.4	Include
Orthopedic Surgery [Wrist Drop Development and Management]	84.4	Include
Orthopedic Surgery [Congenital Deformities of the Extremities and Spine]	84.4	Include
Liver [Hepatic Neoplasms]	83.6	Include
Large Bowel [Colovesical/Colovaginal Fistulas]	83.6	Include
Vascular [Varicose Veins and Chronic Venous Stasis Disease]	83.6	Include
Urology [Carcinoma of the Penis]	83.6	Include
Pancreas [Pancreatic Cysts and Pseudocyst]	82.8	Include
Large Bowel [Clostridium dificille Colitis]	82.8	Include
Endocrine [Adrenal Tumors]	82.8	Include
Endocrine [Hyperparathyroidism]	82.8	Include
Small Intestine [Short Bowel Syndrome]	82.0	Include
Large Bowel [Appendiceal Neoplasm]	82.0	Include
Breast [Phylloides Tumor]	82.0	Include
Urology [Urogenital Fistula]	82.0	Include
Orthopedic Surgery [Metabolic Bone Diseases]	82.0	Include
Neurosurgery [Brain Tumors]	82.0	Include
Others [Introduction to Laparoscopic Surgery]	82.0	Include
Hepatobiliary [Gallstone Ileus]	81.3	Include
Stomach and Duodenum [Gastrointestinal Stromal Tumor]	81.3	Include
Large Bowel [Functional Colonic Disorders]	81.3	Include
Large Bowel [Ischemic Colitis]	81.3	Include
Anorectal [Pilondial Disease]	81.3	Include
Urology [Renal Cysts]	81.3	Include
Abdomen - General / Hernia [Diaphragmatic Hernia]	80.5	Include
Liver [Surgery in the Cirrhotic Patient]	80.5	Include
Large Bowel [Toxic Megacolon]	80.5	Include
Pediatric [Posterior Urethral Valves]	80.5	Include
Pediatric [Congenital Anomalies of External Genitalia]	80.5	Include
Others [The Hidden Curriculum (Non-Explicit Learning from the Surgical Environment)]	79.7	Include
Liver [Benign Hepatic Masses]	78.9	Include
Stomach and Duodenum [Post-gastrectomy Syndromes]	78.9	Include
Endocrine [Parathyroid Tumors]	78.9	Include
Plastic Surgery [Flaps]	78.9	Include
Hepatobiliary [Biliary Surgery]	78.1	Include
Stomach and Duodenum [Gastric Lymphoma]	78.1	Include
Thoracic [Surgical Management of Emphysema]	78.1	Include
Pediatric [Cloaca and Bladder Extrophy]	78.1	Include
Others [Spirituality and Surgery]	78.1	Include
Hepatobiliary [Biliary Duct Injury]	77.3	Include
Liver [Hepatic Biopsy]	77.3	Include
Stomach and Duodenum [Obsesity]	77.3	Include
Esophagus [Benign Esophageal Tumor]	77.3	Include
Endocrine [Multiple Endocrine Neoplasia]	77.3	Include
Hepatobiliary [Cholangiography]	76.6	Include
Stomach and Duodenum [Gastric Volvulus]	76.6	Include
Large Bowel [Colon Carcinoid]	76.6	Include
Esophagus [Esophageal Diverticula]	76.6	Include
Breast [Management of the Axilla in Breast Cancer]	76.6	Include
Urology [Penile Fracture]	76.6	Include
Abdomen - General / Hernia [Management of Abdominal Wall Tumors]	75.8	Include
Abdomen - General / Hernia [Other Herniae (Spigellian, Lumbar, etc.)]	75.8	Include
Esophagus [Esophageal Motility Disorders]	75.8	Include
Thoracic [Mediastinal Tumors]	75.8	Include
Hepatobiliary [Biliary Duct Tumors]	75	Include
Hepatobiliary [Gallbladder Cancer]	75	Include
Small Intestine [Small Intestinal Lymphoma]	75	Include
Esophagus [Paraesophageal Herniae]	75	Include
Orthopedic Surgery [Scoliosis]	75	Include
Pancreas [Management of Complex Pancreatitis]	74.2	Exclude
Small Intestine [Small Intestinal Carcinoid Tumor]	74.2	Exclude
Thoracic [Benign Lung Tumors]	74.2	Exclude
Vascular [Other Aneurysmal Diseases (e.g., Popliteal, Carotid, Pseudoaneurysm)]	74.2	Exclude
Urology [Male Infertility]	73.4	Exclude
Hepatobiliary [Choledochal Cyst]	71.9	Exclude
Hepatobiliary [Sclerosing Cholangitis]	71.9	Exclude
Pancreas [Periampullary Neoplasm]	71.9	Exclude
Small Intestine [Radiation Enteritis]	71.9	Exclude
Pancreas [Pancreatic Fistula]	71.1	Exclude
Stomach and Duodenum [Gastric Polyps]	71.1	Exclude
Vascular [Hemodialysis Access]	71.1	Exclude
Hepatobiliary [Ampullary Stenosis/Sphincter of Oddi Dysfunction]	70.3	Exclude
Liver [Liver Congenital Diseases]	70.3	Exclude
Liver [Budd-Chiari Syndrome]	70.3	Exclude
Hepatobiliary [Gallbladder Polyps]	69.5	Exclude
Spleen [Splenic Tumors]	69.5	Exclude
Abdomen - General / Hernia [Management of Retroperitoneal Tumors]	68.8	Exclude
Breast [Breast Reconstruction]	64.8	Exclude
Others [Transplantation]	63.3	Exclude
Pancreas [Management of Pancreatic Congenital Anomalies (e.g., pancreas divisum)]	60.9	Exclude

Appendix 5: Final modified Delphi round consensus details

Detailed outcomes of the final round are shown in Table 7.

**Table 7:** (Appendix 5) Final Delphi Consensus Result Details Aggregate prioritization score is the sum of assigned scores *Experts in Agreement is the number of experts that rate the item as 'important' (4) or 'somewhat important' (3) ***I-CVI is the proportion of content experts giving item a rating of 'important' (4) or 'somewhat important' (3) **UA assigns 1 to the items that achieve 100% experts in agreement, and 0 if not all experts rate at 'important' (4) or 'somewhat important' (3) ****S-CVI/Ave (based on proportion relevance)

Topic Areas	Aggregate Prioritization Score	Experts in Agreement*	Universal Agreement (UA)**	Item-level content validity index (I-CVI)***	Scale-level content validity index based on the average method (S-CVI/Ave) using proportion relevance)****
Surgery Overview [Wounds and Wound Healing]	100	31	1	1.00	0.935
Surgery Overview [Fluids and Electrolytes in Surgery]	100	31	1	1.00	
Large Bowel [Appendicitis]	100	31	1	1.00	
Pediatric [Intussusception]	99.19354839	31	1	1.00	
Surgery Overview [Physiologic Response to Trauma]	99.19354839	31	1	1.00	
Surgery Overview [Sepsis and Infection]	99.19354839	31	1	1.00	
Surgery Overview [Preoperative Preparation]	99.19354839	31	1	1.00	
Surgery Overview [Antibiotics Use in Surgery]	99.19354839	31	1	1.00	
Surgery Overview [Ethics and Surgery]	99.19354839	31	1	1.00	
Small Intestine [Typhoid Enteritis/Typhoid Ileal Perforation]	99.19354839	31	1	1.00	
Small Intestine [Small Intestinal Obstruction]	99.19354839	31	1	1.00	
Anorectal [Hemorrhoids]	99.19354839	31	1	1.00	
Breast [Breast Infection]	99.19354839	31	1	1.00	
Urology [Acute Urinary Retention ]	99.19354839	31	1	1.00	
Urology [Testicular Torsion]	99.19354839	31	1	1.00	
Surgery Overview [Surgical Quality and Safety]	98.38709677	31	1	1.00	
Surgery Overview [Immediate Postoperative Care]	98.38709677	31	1	1.00	
Stomach and Duodenum [Gastric Outlet Obstruction]	98.38709677	31	1	1.00	
Stomach and Duodenum [Peptic Ulcer]	98.38709677	31	1	1.00	
Stomach and Duodenum [Upper Gastrointestinal Bleeding]	98.38709677	31	1	1.00	
Anorectal [Anal Fissure]	98.38709677	31	1	1.00	
Breast [Breast Cancer]	98.38709677	31	1	1.00	
Pediatric [Fluid and Electrolyte Management]	98.38709677	31	1	1.00	
Urology [Hematuria]	98.38709677	31	1	1.00	
Orthopedic Surgery [Principles of Fracture Management]	98.38709677	31	1	1.00	
Surgery Overview [Classification and Management of Wounds]	97.58064516	31	1	1.00	
Surgery Overview [Imaging in Surgery]	97.58064516	30	0	0.97	
Abdomen - General / Hernia [Management of Acute Abdomen]	97.58064516	31	1	1.00	
Abdomen - General / Hernia [Abdominal Pain]	97.58064516	31	1	1.00	
Large Bowel [Large Bowel Obstruction]	97.58064516	31	1	1.00	
Large Bowel [Lower Gastrointestinal Bleeding]	97.58064516	31	1	1.00	
Thoracic [Thoracic Trauma]	97.58064516	31	1	1.00	
Breast [Breast Diagnostics]	97.58064516	31	1	1.00	
Pediatric [Umbilical Hernia]	97.58064516	31	1	1.00	
Pediatric [Inguinal Hernia]	97.58064516	31	1	1.00	
Pediatric [Approach to Bilious Vomiting in the Newborn]	97.58064516	30	0	0.97	
Urology [Benign Prostate Hypertrophy]	97.58064516	31	1	1.00	
Plastic Surgery [Burns]	97.58064516	31	1	1.00	
Orthopedic Surgery [Approach to Upper Extremity Fractures and Dislocations]	97.58064516	31	1	1.00	
Orthopedic Surgery [Approach to Lower Extremity Fractures and Dislocations]	97.58064516	31	1	1.00	
Orthopedic Surgery [Bone and Joint Infections]	97.58064516	31	1	1.00	
Neurosurgery [Head Trauma]	97.58064516	31	1	1.00	
Others [Responding to Common Perioperative Problems (e.g., fever, tachycardia, chest pain, bleeding)]	97.58064516	31	1	1.00	
Surgery Overview [Surgical Safety Checklist]	96.77419355	31	1	1.00	
Surgery Overview [Nutritional Optimization]	96.77419355	31	1	1.00	
Surgery Overview [Consent for Surgery]	96.77419355	31	1	1.00	
Abdomen - General / Hernia [Inguinal Hernia]	96.77419355	31	1	1.00	
Hepatobiliary [Cholecystitis]	96.77419355	30	0	0.97	
Large Bowel [Volvulus]	96.77419355	30	0	0.97	
Anorectal [Anorectal Abscess and Fistulae]	96.77419355	31	1	1.00	
Esophagus [Dysphagia]	96.77419355	30	0	0.97	
Breast [Evaluation of Breast Pain (Mastalgia, Mastitis)]	96.77419355	30	0	0.97	
Urology [Urethral Stricture]	96.77419355	31	1	1.00	
Plastic Surgery [Necrotizing Soft Tissue Infections]	96.77419355	31	1	1.00	
Others [Surgical Risk Assessment]	96.77419355	31	1	1.00	
Abdomen - General / Hernia [Approach to Blunt and Penetrating Abdominal Trauma]	95.96774194	30	0	0.97	
Spleen [Splenomegaly]	95.96774194	31	1	1.00	
Small Intestine [Paralytic Ileus]	95.96774194	30	0	0.97	
Esophagus [Gastro-esophageal Reflux]	95.96774194	31	1	1.00	
Vascular [Deep Vein Thrombosis and Thromboembolic Disease]	95.96774194	31	1	1.00	
Breast [Benign Breast Disease]	95.96774194	31	1	1.00	
Pediatric [Anorectal Malformations]	95.96774194	30	0	0.97	
Pediatric [Nutrition]	95.96774194	31	1	1.00	
Urology [Prostatitis and Epididymitis]	95.96774194	30	0	0.97	
Others [Acute Airway Obstruction in Adult and Pediatric Patients]	95.96774194	31	1	1.00	
Surgery Overview [Communication in Surgery]	95.16129032	30	0	0.97	
Hepatobiliary [Obstructive Jaundice]	95.16129032	30	0	0.97	
Liver [Liver Abscesses]	95.16129032	30	0	0.97	
Spleen [Management of Splenic Trauma]	95.16129032	31	1	1.00	
Vascular [Peripheral Arterial Disease]	95.16129032	30	0	0.97	
Pediatric [Undescended Testes]	95.16129032	31	1	1.00	
Urology [Renal Trauma]	95.16129032	31	1	1.00	
Orthopedic Surgery [Diabetic Foot Ulcers]	95.16129032	31	1	1.00	
Orthopedic Surgery [Extremity Compartment Syndrome]	95.16129032	30	0	0.97	
Others [Acute and Chronic Pain Management]	95.16129032	30	0	0.97	
Hepatobiliary [Cholangitis]	94.35483871	30	0	0.97	
Liver [Hepatic Injury]	94.35483871	30	0	0.97	
Pancreas [Acute Pancreatitis]	94.35483871	30	0	0.97	
Breast [Breast Carcinoma In-Situ]	94.35483871	31	1	1.00	
Pediatric [Pyloric Stenosis]	94.35483871	31	1	1.00	
Pediatric [Circumscision]	94.35483871	30	0	0.97	
Urology [Urolithiasis]	94.35483871	31	1	1.00	
Orthopedic Surgery [Approach to Pelvic and Sacral Fractures]	94.35483871	31	1	1.00	
Orthopedic Surgery [Approach to Spine Fractures]	94.35483871	31	1	1.00	
Others [Feeding Access (Tubes, Lines, and Drains)]	94.35483871	31	1	1.00	
Abdomen - General / Hernia [Femoral Hernia]	93.5483871	31	1	1.00	
Hepatobiliary [Choledocholithiasis]	93.5483871	30	0	0.97	
Large Bowel [Colon and Rectal Cancer]	93.5483871	30	0	0.97	
Large Bowel [Colostomy]	93.5483871	30	0	0.97	
Thoracic [Pulmonary and Pleural TB]	93.5483871	30	0	0.97	
Endocrine [Goitre]	93.5483871	29	0	0.94	
Pediatric [Gastroschisis and Omphalocoele]	93.5483871	30	0	0.97	
Urology [Urologic Trauma (including iatrogenic)]	93.5483871	31	1	1.00	
Abdomen - General / Hernia [Incisional Hernia]	92.74193548	30	0	0.97	
Abdomen - General / Hernia [Abdominal Compartment Syndrome]	92.74193548	30	0	0.97	
Stomach and Duodenum [Gastric Cancer]	92.74193548	30	0	0.97	
Breast [Role of Community Health Workers in Prevention, Screening, and Community Sensitization of Breast Disease]	92.74193548	30	0	0.97	
Pediatric [Necrotizing Enterocolitis]	92.74193548	29	0	0.94	
Pediatric [Venous Access]	92.74193548	29	0	0.94	
Urology [Prostate Cancer]	92.74193548	31	1	1.00	
Urology [Varicocele]	92.74193548	31	1	1.00	
Orthopedic Surgery [Swollen/Painful Knee]	92.74193548	29	0	0.94	
Neurosurgery [Surgical CNS Infections]	92.74193548	30	0	0.97	
Pancreas [Chronic Pancreatitis]	91.93548387	30	0	0.97	
Breast [Paget's Disease]	91.93548387	28	0	0.90	
Orthopedic Surgery [Tuberculosis of the Spine]	91.93548387	31	1	1.00	
Neurosurgery [Acute Stroke Care]	91.93548387	29	0	0.94	
Others [Patient Positioning]	91.93548387	30	0	0.97	
Surgery Overview [Non-technical Skills for Surgery]	91.12903226	31	1	1.00	
Surgery Overview [Outpatient Follow-up of Common Surgical Conditions and Long-Term Outcomes]	91.12903226	30	0	0.97	
Abdomen - General / Hernia [Differential Diagnosis of a Right Iliac Fossa Mass]	91.12903226	29	0	0.94	
Hepatobiliary [Biliary Disease Evaluation]	91.12903226	29	0	0.94	
Small Intestine [Short Bowel Syndrome]	91.12903226	28	0	0.90	
Large Bowel [Constipation]	91.12903226	28	0	0.90	
Anorectal [Rectal Cancer]	91.12903226	29	0	0.94	
Anorectal [Rectal Prolapse]	91.12903226	29	0	0.94	
Esophagus [esophageal Stricture]	91.12903226	30	0	0.97	
Thoracic [Surgical Lung Infections (Lung Abscess, Bronchiectasis, Empyema)]	91.12903226	29	0	0.94	
Vascular [Central Venous Access]	91.12903226	29	0	0.94	
Vascular [Varicose Veins and Chronic Venous Stasis Disease]	91.12903226	28	0	0.90	
Endocrine [Thyroid Cancers]	91.12903226	29	0	0.94	
Urology [Testicular Tumor]	91.12903226	31	1	1.00	
Plastic Surgery [Approach to Hand Infections]	91.12903226	29	0	0.94	
Spleen [Post Splenectomy Syndrome]	90.32258065	30	0	0.97	
Small Intestine [Mesenteric Ischaemia]	90.32258065	29	0	0.94	
Esophagus [Esophageal Foreign Body]	90.32258065	30	0	0.97	
Endocrine [Thyroid Nodules]	90.32258065	29	0	0.94	
Endocrine [Graves Disease]	90.32258065	29	0	0.94	
Plastic Surgery [Approach to Hand Injuries]	90.32258065	29	0	0.94	
Orthopedic Surgery [Amputations and Disarticulations]	90.32258065	28	0	0.90	
Orthopedic Surgery [Carpal Tunnel Syndrome]	90.32258065	29	0	0.94	
Neurosurgery [Hydrocephalus]	90.32258065	29	0	0.94	
Hepatobiliary [Biliary Pancreatitis]	89.51612903	29	0	0.94	
Liver [Portal Hypertension]	89.51612903	29	0	0.94	
Esophagus [Barrett's Esophagus]	89.51612903	29	0	0.94	
Esophagus [Esophageal Cancer]	89.51612903	30	0	0.97	
Urology [Incontinence]	89.51612903	29	0	0.94	
Others [Reflective Writing (medical student journaling to generate compassion for patients)]	89.51612903	31	1	1.00	
Others [HIV/AIDS and the Surgical Patient]	89.51612903	31	1	1.00	
Others [Surgical Audit and Research]	89.51612903	30	0	0.97	
Surgery Overview [Introduction to Global Surgery]	88.70967742	30	0	0.97	
Liver [Liver Hydatid Dieseases]	88.70967742	29	0	0.94	
Stomach and Duodenum [Stress Gastritis]	88.70967742	29	0	0.94	
Esophagus [Esophageal Perforation]	88.70967742	28	0	0.90	
Endocrine [Thyroiditis]	88.70967742	30	0	0.97	
Pediatric [Tracheo-esphogeal Congenital Anomolies]	88.70967742	28	0	0.90	
Orthopedic Surgery [Cord Compression Syndromes]	88.70967742	29	0	0.94	
Others [Difficult Patient Encounters]	88.70967742	29	0	0.94	
Surgery Overview [Introduction to Surgery/History of Surgery]	87.90322581	30	0	0.97	
Liver [Differential Diagnosis of Hepatic Masses]	87.90322581	27	0	0.87	
Spleen [Splenic Abscess]	87.90322581	31	1	1.00	
Small Intestine [Meckel's Diverticulum]	87.90322581	29	0	0.94	
Large Bowel [Inflammatory Bowel Disease (Ulcerative Colitis and Crohn's Disease)]	87.90322581	27	0	0.87	
Vascular [Abdominal Aortic Aneurysm]	87.90322581	29	0	0.94	
Vascular [Vascular Trauma]	87.90322581	29	0	0.94	
Endocrine [Pheochromocytoma]	87.90322581	28	0	0.90	
Pediatric [Gastrointestinal Tract Atresia and Stenosis]	87.90322581	29	0	0.94	
Plastic Surgery [Skin Grafts]	87.90322581	28	0	0.90	
Orthopedic Surgery [Tropical Leg Ulcers]	87.90322581	29	0	0.94	
Small Intestine [Gastrointestinal Fistula (Enterocutaneous and Enteroatmospheric)]	87.09677419	28	0	0.90	
Large Bowel [Malignant Bowel Obstruction]	87.09677419	27	0	0.87	
Large Bowel [Ischemic Colitis]	87.09677419	28	0	0.90	
Vascular [Lymphedema]	87.09677419	29	0	0.94	
Breast [Gynecomastia]	87.09677419	28	0	0.90	
Orthopedic Surgery [Painful/Paralyzed Limb]	87.09677419	27	0	0.87	
Orthopedic Surgery [Volksman Ischemic Contracture]	87.09677419	29	0	0.94	
Stomach and Duodenum [Gastric Volvulus]	86.29032258	28	0	0.90	
Large Bowel [Clostridium dificille Colitis]	86.29032258	28	0	0.90	
Anorectal [Anal Cancer]	86.29032258	27	0	0.87	
Breast [Phylloides Tumour]	86.29032258	28	0	0.90	
Breast [Management of the Axilla in Breast Cancer]	86.29032258	27	0	0.87	
Endocrine [Hyperparathyroidism]	86.29032258	29	0	0.94	
Pediatric [Surgical Jaundice in the Newborn]	86.29032258	28	0	0.90	
Pediatric [Hypospadias and Epispadias]	86.29032258	28	0	0.90	
Pediatric [Posterior Urethral Valves]	86.29032258	28	0	0.90	
Urology [Carcinoma of the Penis]	86.29032258	28	0	0.90	
Urology [Penile Fracture]	86.29032258	28	0	0.90	
Plastic Surgery [Skin Tumors]	86.29032258	30	0	0.97	
Plastic Surgery [Keloids and Hypertrophic Scars]	86.29032258	29	0	0.94	
Orthopedic Surgery [Wrist Drop Development and Management]	86.29032258	28	0	0.90	
Abdomen - General / Hernia [Management of Abdominal Wall Tumors]	85.48387097	28	0	0.90	
Hepatobiliary [Parasitic Biliary Obstruction]	85.48387097	28	0	0.90	
Pancreas [Pancreatic Neoplasm]	85.48387097	27	0	0.87	
Spleen [Splenectomy]	85.48387097	26	0	0.84	
Stomach and Duodenum [Postgastrectomy Syndromes]	85.48387097	27	0	0.87	
Thoracic [Lung Cancer]	85.48387097	31	1	1.00	
Vascular [Arterial Access]	85.48387097	26	0	0.84	
Breast [Breast Sarcoma]	85.48387097	29	0	0.94	
Pediatric [Congenital Anomalies of External Genitalia]	85.48387097	27	0	0.87	
Orthopedic Surgery [Bone Tumors]	85.48387097	30	0	0.97	
Pancreas [Pancreatic Cysts and Pseudocyst]	84.67741935	25	0	0.81	
Large Bowel [Colon Polyps]	84.67741935	28	0	0.90	
Anorectal [Fecal Incontinence]	84.67741935	27	0	0.87	
Endocrine [Adrenal Tumors]	84.67741935	29	0	0.94	
Pediatric [Pediatric Pleural Infections]	84.67741935	25	0	0.81	
Urology [Bladder Tumors]	84.67741935	29	0	0.94	
Urology [Renal Tumor]	84.67741935	28	0	0.90	
Others [Same-Day Surgery]	84.67741935	29	0	0.94	
Hepatobiliary [Gallstone Ileus]	83.87096774	28	0	0.90	
Large Bowel [Diverticular Disease]	83.87096774	27	0	0.87	
Urology [Urogenital Fistula]	83.87096774	26	0	0.84	
Neurosurgery [Neural Tube Defects]	83.87096774	28	0	0.90	
Abdomen - General / Hernia [Diaphragmatic Hernia]	83.06451613	28	0	0.90	
Liver [Hepatic Neoplasms]	83.06451613	27	0	0.87	
Liver [Parasitic Liver Diseases of Surgical Importance]	83.06451613	27	0	0.87	
Stomach and Duodenum [Gastrointestinal Stromal Tumor]	83.06451613	28	0	0.90	
Small Intestine [Small Intestinal Lymphoma]	83.06451613	28	0	0.90	
Anorectal [Condyloma]	83.06451613	26	0	0.84	
Esophagus [Paraesophageal Herniae]	83.06451613	27	0	0.87	
Vascular [Other Aneurysmal Diseases (e.g., Popliteal, Carotid, Pseudoaneurysm)]	83.06451613	26	0	0.84	
Endocrine [Parathyroid Tumors]	83.06451613	28	0	0.90	
Plastic Surgery [Cleft Lip and Palate]	83.06451613	28	0	0.90	
Liver [Benign Hepatic Masses]	82.25806452	25	0	0.81	
Esophagus [Benign esophageal Tumor]	82.25806452	27	0	0.87	
Thoracic [Surgical Management of Emphysema]	82.25806452	25	0	0.81	
Pediatric [Pediatric Neoplasms (e.g., Neuroblastoma, Nephroblastoma)]	82.25806452	26	0	0.84	
Orthopedic Surgery [Congenital Deformities of the Extremities and Spine]	82.25806452	28	0	0.90	
Others [Surgery in the Elderly]	82.25806452	27	0	0.87	
Abdomen - General / Hernia [Other Herniae (Spigellian, Lumbar, etc.)]	81.4516129	27	0	0.87	
Large Bowel [Colovesical/Colovaginal Fistulas]	81.4516129	26	0	0.84	
Large Bowel [Functional Colonic Disorders]	81.4516129	26	0	0.84	
Esophagus [esophageal Diverticula]	81.4516129	26	0	0.84	
Esophagus [esophageal Motility Disorders]	81.4516129	27	0	0.87	
Pediatric [Cloaca and Bladder Extrophy]	81.4516129	27	0	0.87	
Orthopedic Surgery [Scoliosis]	81.4516129	27	0	0.87	
Others [Spirituality and Surgery]	81.4516129	24	0	0.77	
Large Bowel [Toxic Megacolon]	80.64516129	27	0	0.87	
Anorectal [Pilondial Disease]	80.64516129	28	0	0.90	
Urology [Renal Cysts]	80.64516129	25	0	0.81	
Hepatobiliary [Cholangiography]	79.83870968	26	0	0.84	
Stomach and Duodenum [Gastric Lymphoma]	79.83870968	26	0	0.84	
Large Bowel [Appendiceal Neoplasm]	79.83870968	26	0	0.84	
Thoracic [Mediastinal Tumors]	79.83870968	25	0	0.81	
Orthopedic Surgery [Metabolic Bone Diseases]	79.83870968	28	0	0.90	
Others [The Hidden Curriculum (Non-Explicit Learning from the Surgical Environment)]	79.83870968	28	0	0.90	
Stomach and Duodenum [Obesity]	79.03225806	25	0	0.81	
Endocrine [Multiple Endocrine Neoplasia]	79.03225806	25	0	0.81	
Plastic Surgery [Flaps]	79.03225806	25	0	0.81	
Others [Introduction to Laparoscopic Surgery]	79.03225806	26	0	0.84	
Hepatobiliary [Biliary Duct Tumors]	78.22580645	25	0	0.81	
Large Bowel [Colon Carcinoid]	78.22580645	27	0	0.87	
Neurosurgery [Brain Tumours]	78.22580645	26	0	0.84	
Hepatobiliary [Biliary Duct Injury]	77.41935484	22	0	0.71	
Hepatobiliary [Gallbladder Cancer]	76.61290323	23	0	0.74	
Liver [Surgery in the Cirrhotic Patient]	76.61290323	24	0	0.77	
Liver [Hepatic Biopsy]	75.80645161	25	0	0.81	
Hepatobiliary [Biliary Surgery]	71.77419355	23	0	0.74	

## References

[1] Meara JG, Leather AJ, Hagander L (2015). Global surgery 2030: evidence and solutions for achieving health, welfare, and economic development. Lancet.

[2] O'Flynn E, Andrew J, Hutch A (2016). The specialist surgeon workforce in East, Central and Southern Africa: a situation analysis. World J Surg.

[3] Meara JG, Greenberg SL (2015). The Lancet Commission on Global Surgery Global surgery 2030: evidence and solutions for achieving health, welfare and economic development. Surgery.

[4] (2023). COSECSA: College of Surgeons of East, Central and Southern Africa strategic plan 2021-2025. http://www.cosecsa.org/wp-content/uploads/2021/01/COSECSA-Strategic-Plan-2021-2025-1.pdf.

[5] Rickard J, Ssebuufu R, Kyamanywa P, Ntakiyiruta G (2016). Scaling up a surgical residency program in Rwanda. East Centr Afr J Surg.

[6] Van Essen C, Steffes BC, Thelander K, Akinyi B, Li HF, Tarpley MJ (2019). Increasing and retaining African surgeons working in rural hospitals: an analysis of PAACS surgeons with twenty-year program follow-up. World J Surg.

[7] (2018). National Surgical Obstetrics and Anesthesia Plan 2018-2024. https://www.google.com/url?sa=t&rct=j&q=&esrc=s&source=web&cd=&cad=rja&uact=8&ved=2ahUKEwio1eKXmf__AhUISEEAHdzvDw0QFnoECBIQAQ&url=https%3A%2F%2Fwww.moh.gov.rw%2Ffileadmin%2Fuser_upload%2FMoh%2FPublications%2FStrategic_Plan%2FNSOAP_Rwanda-_Approved1.pdf&usg=AOvVaw0T3H3NHV9A0Vn9Xu3lm5SJ&opi=89978449.

[8] Maine RG, Linden AF, Riviello R (2017). Prevalence of untreated surgical conditions in rural Rwanda: a population-based cross-sectional study in Burera District. JAMA Surg.

[9] Galukande M, Kaggwa S, Sekimpi P (2013). Use of surgical task shifting to scale up essential surgical services: a feasibility analysis at facility level in Uganda. BMC Health Serv Res.

[10] Ashley T, Ashley H, Wladis A (2021). Outcomes after elective inguinal hernia repair performed by associate clinicians vs medical doctors in Sierra Leone: a randomized clinical trial. JAMA Netw Open.

[11] Dworkin M, Nsengimana V, Rosenberg A (2020). Prehospital epidemiology and management of injured children in Kigali, Rwanda. Emerg Med J.

[12] Mpirimbanyi C, Nyirimodoka A, Lin Y (2017). Emergency general surgery in Rwandan district hospitals: a cross-sectional study of spectrum, management, and patient outcomes. BMC Surg.

[13] Falk R, Taylor R, Kornelsen J, Virk R (2020). Surgical task-sharing to non-specialist physicians in low-resource settings globally: a systematic review of the literature. World J Surg.

[14] Livergant RJ, Demetrick S, Cravetchi X, Kung JY, Joos E, Hawes HG, Saleh A (2021). Trauma training courses and programs in low- and lower middle-income countries: a scoping review. World J Surg.

[15] Bekele A, Wong R (2019). University of Global Health Equity - a new way of institutional higher education. J Management Strategy.

[16] O'Neill KM, Greenberg SL, Cherian M (2016). Bellwether procedures for monitoring and planning essential surgical care in low- and middle-income countries: caesarean delivery, laparotomy, and treatment of open fractures. World J Surg.

[17] Nsanzabaganwa C, Habineza H, Nyirimanzi N, Umuhoza C, Cartledge K, Conard C, Cartledge P (2019). Write-up and dissemination of undergraduate and postgraduate research at the University of Rwanda: a cross-sectional study. Pan Afr Med J.

[18] Barrett D, Heale R (2020). What are Delphi studies?. Evid Based Nurs.

[19] Hsieh CJ, Fifić M, Yang CT (2020). A new measure of group decision-making efficiency. Cogn Res Princ Implic.

[20] Okoli C, Pawlowski SD (2004). The Delphi method as a research tool: an example, design considerations and applications. Inf Manag.

[21] Salmon G, Tombs M (2018). Teaching undergraduate medical students Child and Adolescent Psychiatry (CAP): a Delphi study on curriculum content. BMC Med Educ.

[22] Alahlafi A, Burge S (2005). What should undergraduate medical students know about psoriasis? Involving patients in curriculum development: modified Delphi technique. BMJ.

[23] Walley T, Webb DJ (1997). Developing a core curriculum in clinical pharmacology and therapeutics: a Delphi study. Br J Clin Pharmacol.

[24] Jünger S, Payne SA, Brine J, Radbruch L, Brearley SG (2017). Guidance on Conducting and REporting DElphi Studies (CREDES) in palliative care: recommendations based on a methodological systematic review. Palliat Med.

[25] Akins RB, Tolson H, Cole BR (2005). Stability of response characteristics of a Delphi panel: application of bootstrap data expansion. BMC Med Res Methodol.

[26] (2023). Organization of African Unity council of ministers. Resolutions of the twenty-sixth ordinary session of the council of ministers. AHG/Res. 453 - 472 (XXVI). https://au.int/sites/default/files/decisions/9591-council_en_23_february_1_march_1976_council_ministers_twenty_sixth_ordinary_session.pdf.

[27] Yusoff MS (2019). ABC of content validation and content validity index calculation. Educ Med J.

[28] Lynn MR (1986). Determination and quantification of content validity. Nurs Res.

[29] Diamond IR, Grant RC, Feldman BM, Pencharz PB, Ling SC, Moore AM, Wales PW (2014). Defining consensus: a systematic review recommends methodologic criteria for reporting of Delphi studies. J Clin Epidemiol.

[30] Hartnett T (2023). Group Facilitation. The Basics of Consensus Decision Making. Hartnett T. https://www.groupfacilitation.net/Articles%20for%20Facilitators/The%20Basics%20of%20Consensus%20Decision%20Making.pdf.

[31] Hennus MP, Jarrett JB, Taylor DR, Ten Cate O (2023). Twelve tips to develop entrustable professional activities. Med Teach.

[32] Ten Cate O (2013). Nuts and bolts of entrustable professional activities. J Grad Med Educ.

[33] Bentounsi Z, Sheik-Ali S, Drury G, Lavy C (2021). Surgical care in district hospitals in sub-Saharan Africa: a scoping review. BMJ Open.

[34] van Heemskerken P, Broekhuizen H, Gajewski J, Brugha R, Bijlmakers L (2020). Barriers to surgery performed by non-physician clinicians in sub-Saharan Africa-a scoping review. Hum Resour Health.

[35] Spratt JS, Papp KK (1997). Practicing primary care physicians’ perspectives on the junior surgical clerkship. Am J Surg.

[36] Selby LV, Coleman JR, Jones TS, Nehler M, Montero P (2021). Surgeons underestimate the importance of surgical topics for non-surgeons: results of a national survey. J Surg Educ.

[37] Teichman JM, Weiss BD, Solomon D (1999). Urological needs assessment for primary care practice: implications for undergraduate medical education. J Urol.

[38] Held MF, Laubscher M, Graham SM, Kruger N, Njisane P, Njisane V, Dunn RN (2020). Topics, skills, and cases for an undergraduate musculoskeletal curriculum in southern Africa: a consensus from local and international experts. J Bone Joint Surg Am.

